# Laparoscopic Cytoreduction After Neoadjuvant Chemotherapy in High-Grade Epithelial Ovarian Cancer: A LANCE Randomized Clinical Trial

**DOI:** 10.1001/jamanetworkopen.2024.46325

**Published:** 2024-11-21

**Authors:** J. Alejandro Rauh-Hain, Alexander Melamed, René Pareja, Taymaa May, Abdulrahman Sinno, Leah McNally, Neil S. Horowitz, Pierandrea De Iaco, Chad M. Michener, Luc Van Lonkhuijzen, Maria D. Iniesta, Ying Yuan, Pedro T. Ramirez, Anna Fagotti

**Affiliations:** 1 Department of Gynecologic Oncology and Reproductive Medicine, The University of Texas MD Anderson Cancer Center, Houston; 2 Department of Health Services Research, The University of Texas MD Anderson Cancer Center, Houston; 3 Division of Gynecologic Oncology, Vincent Obstetrics and Gynecology, Massachusetts General Hospital, Harvard Medical School, Boston; 4 Department of Gynecologic Oncology, Instituto Nacional de Cancerología, Bogotá, Colombia; 5 Princess Margaret Hospital Cancer Centre, Toronto, Ontario, Canada; 6 Division of Gynecologic Oncology, Department of Medical Oncology, Dana-Farber Cancer Institute, Boston, Massachusetts; 7 Department of Obstetrics and Gynaecology, University of Toronto, Toronto, Ontario, Canada; 8 Department of Obstetrics and Gynecology, Division of Gynecological Oncology, University of Miami Miller School of Medicine, Miami, Florida; 9 Duke University, Durham, North Carolina; 10 Division of Oncologic Gynecology, Istituto di Ricovero e Cura a Carattere Scientifico, Azienda Ospedaliero-Universitaria di Bologna, Bologna, Italy; 11 Obstetrics and Gynecology Institute, Cleveland Clinic, Cleveland, Ohio; 12 Department of Gynecologic Oncology, Amsterdam University Medical Center, Center for Gynecological Oncology Amsterdam, Amsterdam, the Netherlands; 13 Department of Biostatistics, The University of Texas MD Anderson Cancer Center, Houston; 14 Department of Obstetrics and Gynecology, Houston Methodist Hospital, Neal Cancer Center, Houston, Texas; 15 Department of Women’s and Children’s Health, Policlinico A Gemelli, Rome, Italy

## Abstract

**Importance:**

Despite the absence of high-quality evidence of its safety and effectiveness, minimally invasive surgery (MIS) is increasingly used to treat advanced epithelial ovarian cancer (EOC).

**Objective:**

To assess the feasibility of conducting a full-scale randomized clinical trial (RCT) designed to compare the efficacy of MIS vs laparotomy in patients with advanced-stage EOC and a complete or partial response to neoadjuvant chemotherapy (NACT).

**Design, Setting, and Participants:**

This lead-in pilot phase of LANCE (Laparoscopic Cytoreduction After Neoadjuvant Chemotherapy), an international, open-label, noninferiority RCT, opened to enrollment in September 2020 and enrolled the 100th eligible patient in February 2023. It was conducted at 11 academic cancer centers in North America and Europe. Participants were adults with stage IIIC or IV epithelial ovarian, fallopian, or primary peritoneal carcinoma who had normal cancer antigen 125 levels and at least a partial radiologic response after 3 to 4 cycles of NACT. Patients were randomly assigned to receive either interval cytoreductive surgery performed using MIS or laparotomy. Data analysis was based on the evaluable population.

**Interventions:**

The MIS arm underwent laparoscopic or robotic surgery, vs laparotomy for the control arm. Resection of all visible tumor was attempted, and conversion from MIS to laparotomy was performed when necessary to attain complete resection.

**Main Outcomes and Measures:**

Trial feasibility was defined by 3 primary end points: patient accrual rate of at least 5.6 patients per month by the last month of the lead-in pilot phase, conversion from MIS to laparotomy in less than 25% of patients, and a difference in complete gross resection rates of fewer than 20 percentage points between study arms.

**Results:**

One hundred women (median [IQR] age, 63 [39-82] years) were included, of whom 49 were randomly assigned to MIS and 51 to laparotomy; 95 were evaluable for surgical outcomes. Most patients (34 [67%] in the laparotomy arm, and 33 [67%] in the MIS arm) had stage IIIC cancer. The monthly accrual rate reached 5.9 patients per month in the final month of the study. Six of 48 evaluable patients (12.5%; 95% CI, 4.7%-25.2%) assigned to MIS underwent conversion to laparotomy. Surgeons achieved a complete gross resection rate in 42 of 48 evaluable patients (88%) assigned to MIS and in 39 of 47 patients (83%) assigned to laparotomy (difference, 4.5 [95% CI, −9.7 to 18.8] percentage points).

**Conclusions and Relevance:**

Results of this lead-in pilot study indicated the feasibility of the LANCE RCT to compare the oncological outcomes of MIS vs laparotomy.

**Trial Registration:**

ClinicalTrials.gov Identifier: NCT04575935

## Key Points

**Question:**

Is it feasible to conduct a phase 3 multicenter randomized clinical trial (RCT) comparing the oncological efficacy of minimally invasive surgery (MIS) with that of laparotomy for interval cytoreductive surgery among patients with high-grade epithelial cancer of the ovary, fallopian tube, or peritoneum?

**Findings:**

In this lead-in pilot phase of an RCT involving 100 women randomly assigned to receive MIS or laparotomy, the predefined patient accrual rate, low frequency of conversion from MIS to laparotomy, and similar complete gross resection rates were observed.

**Meaning:**

Findings of this study suggest that a full-scale RCT comparing the oncological efficacy of MIS vs laparotomy is feasible.

## Teaser

This randomized clinical trial compares patient accrual rate, treatment conversion rate, complete gross resection rate, and other feasibility outcomes of a minimally invasive surgical approach vs laparotomy in patients with advanced-stage epithelial ovarian cancer.

## Introduction

Cytoreductive surgery, either as the primary treatment or after neoadjuvant chemotherapy (NACT), for advanced epithelial ovarian cancer (EOC), fallopian tube carcinoma, and primary peritoneal cancer carries a substantial risk of complications^1^ that may hinder the timely administration of chemotherapy,^2^ lead to prolonged hospital stays and readmissions,^3^ and decrease survival.^4^ Although primary cytoreductive surgery followed by platinum-based adjuvant chemotherapy has been the standard-of-care treatment for advanced-stage EOC,^5,6,7^randomized clinical trials (RCTs) have demonstrated that administering NACT before cytoreductive surgery can reduce surgical morbidity and improve patients’ quality of life without compromising overall survival.^8,9,10,11,12,13,14^ Among patients who receive NACT, interval cytoreductive surgery performed with minimally invasive surgical techniques may further reduce morbidity.^15,16,17,18,19,20,21,22^ As the use of NACT has become increasingly common in the management of advanced-stage EOC,^23,24^ interval cytoreductive surgeries performed with minimally invasive techniques have also increased. By 2018, 29% of all interval cytoreductive surgeries conducted in Commission on Cancer–accredited cancer programs used a minimally invasive approach.^24,25^

The existing observational data suggest that in women with advanced EOC who have had a complete or almost complete response to NACT, minimally invasive surgery (MIS) is associated with high rates of complete cytoreduction, excellent progression-free survival, and long-term overall survival similar to that in patients who undergo laparotomy.^15,16,17,18,19,20,21,25^ Nevertheless, despite the growing adoption of minimally invasive interval cytoreductive surgery, the current body of evidence remains inconclusive because observational studies tend to be biased. The importance of RCTs evaluating the efficacy of minimally invasive cancer surgery is highlighted by the recent experiences in cervical cancer treatment, in which minimally invasive radical hysterectomy, once considered a standard approach solely based on retrospective observational individual-level studies, resulted in higher rates of cervical cancer recurrence and mortality compared with open radical hysterectomy.^26,27^

The Laparoscopic Cytoreduction After Neoadjuvant Chemotherapy (LANCE) trial is an international, open-label, noninferiority RCT designed to compare the efficacy of MIS vs laparotomy among patients with stage IIIC or IV high-grade EOC and a complete or partial response to NACT. The results of the lead-in pilot phase reported here aimed to assess the feasibility of conducting a full-scale RCT.

## Methods

Details regarding the LANCE trial have been previously reported.^28^ The first 100 patients randomized were included in this lead-in pilot phase of the trial. This trial was conducted between September 2020 and February 2023 at 11 academic cancer centers in North America and Europe. The University of Texas MD Anderson Cancer Center Institutional Review Board approved this RCT, and all participants provided written informed consent. The trial protocol (Supplement 1) was registered a priori. We followed the Consolidated Standards of Reporting Trials (CONSORT) reporting guideline.

### Population

Patients were included if they met the following criteria: (1) aged 18 years or older with stage IIIC or IV, high-grade serous, endometrioid, clear cell, or transitional invasive EOC (including primary peritoneal and fallopian tube carcinomas); (2) considered by the treating physician to be a candidate for surgery after the administration of 3 to 4 cycles of NACT, with complete radiologic resolution of any disease outside the abdominal cavity (pleural effusions were acceptable per the local principal investigator’s discretion); (3) normalization of cancer antigen 125 (CA-125) levels according to the participating center’s reference range; (4) time frame of less than 6 weeks from the last cycle of NACT to interval cytoreductive surgery; and (5) Eastern Cooperative Oncology Group Performance Status score of 0 to 2 (score range: 0-5, with the highest score indicating death). Patients were excluded if preoperative imaging showed tumors that the surgeon determined to be unsuitable for minimally invasive resection or if there was any contraindication to MIS.

The lead-in pilot phase of the LANCE trial began enrollment in September 2020, and the 100th eligible patient was enrolled in February 2023 across 11 centers (8 in North America, and 3 in Europe). The following criteria were used to screen centers for participation: (1) treatment of at least 30 patients with advanced-stage EOC every year, (2) existence of a multidisciplinary tumor board with at least 1 surgeon and 1 pathologist, (3) availability of chemotherapy, and (4) experience of the team working in RCTs.

### Intervention

**Figure 1.**
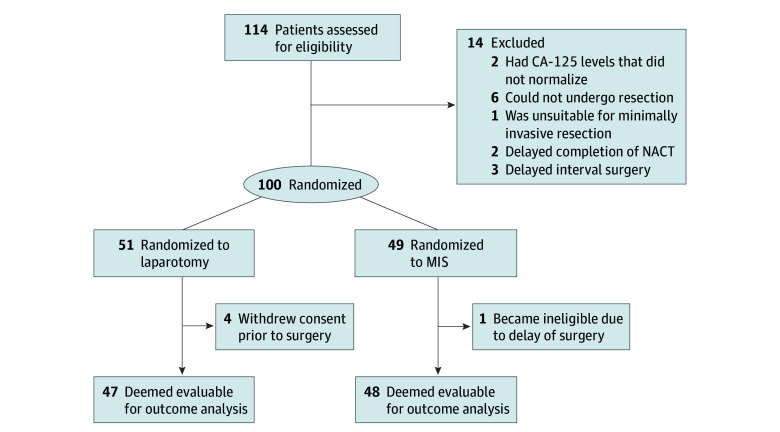
Flow Diagram of Patient Selection and Randomization CA-125 indicates cancer antigen 125; MIS, minimally invasive surgery; NACT, neoadjuvant chemotherapy.

Patients were randomly assigned to receive either interval cytoreductive surgery performed using MIS (traditional or robot-assisted laparoscopy; intervention arm) or laparotomy (control arm) (Figure 1). Randomization was performed using a minimization algorithm to balance disease stage, *BRCA* status, and planned use of hyperthermic intraperitoneal chemotherapy between the 2 arms.

Surgery was performed as soon as possible after hematologic recovery and no more than 6 weeks after the final cycle of NACT. Every surgeon sought to resect all visible tumors, but the technical aspects of each surgery, such as instrumentation and location of incisions, were left to the surgeon’s discretion. Among patients assigned to MIS, conversion to laparotomy was recommended when a surgeon determined that conversion would facilitate a complete resection that could not be achieved using the MIS approach. Details on the planned and actual surgeries were collected from baseline and postoperative case report forms. A Surgeon Evaluation Committee assesses the competence of each surgeon to perform the surgical interventions required by the LANCE trial. Surgeons must have performed a minimum of 25 cancer cases using MIS in the past year, with at least 5 of these procedures involving ovarian cancer. Additionally, participating surgeons are required to submit 2 unedited videos of themselves performing a minimally invasive interval debulking procedure. These videos are formally assessed by the committee to ensure proficiency in the procedure.

The recommended NACT regimen was paclitaxel (175 mg/m^2^ of body surface) administered as a 3-hour infusion, immediately followed by an intravenous infusion of carboplatin (area under the curve = 6) over 1 hour administered every 3 weeks with or without bevacizumab 15 mg/kg. However, the intended regimen was established on an individual basis, and the patient’s fitness and choice were taken into consideration. Regimens and combinations other than the recommended NACT regimen were allowed as long as they were considered standard-of-care treatments or were part of an approved research protocol. Patients could receive 3 or 4 cycles of chemotherapy before cytoreductive surgery and were required to receive at least 6 cycles in total. The addition of hyperthermic intraperitoneal chemotherapy at the time of interval cytoreductive surgery was allowed if the surgical team was able to perform this procedure with either MIS or laparotomy.^29^ The time to resumption of chemotherapy after surgery was determined by the treating oncologist but recommended within 6 weeks of surgery. Patients received maintenance therapy with bevacizumab and/or poly(adenosine diphosphate–ribose) polymerase inhibitors as clinically indicated.

### Outcomes

The main objective of this phase of the trial was to establish the feasibility of an RCT. Feasibility was defined by 3 primary end points: patient accrual rate of at least 5.6 patients per month (80% of the target rate of 7 patients per month) by the last month of the lead-in phase, conversion to laparotomy in less than 25% of patients in the MIS arm, and a difference in the complete gross resection rates between the laparotomy and MIS arms of less than 20 percentage points.

The secondary outcomes included the length of postoperative hospitalization, estimated blood loss, intraoperative complications, postoperative complications within 30 days after surgery, time to resumption of chemotherapy, and health-related quality of life (HRQOL). Intraoperative and postoperative adverse events were graded using the Common Terminology Criteria for Adverse Events (CTCAE), version 5.0 (grades 1 [indicating asymptomatic or mild] to 4 [indicating life-threatening]), and/or the Clavien-Dindo classification (grades 1 [indicating any deviation from the normal postoperative course without the need for pharmacological treatment or surgical, endoscopic, and radiological interventions] to 5 [indicating death]). HRQOL was assessed at baseline, the first postoperative visit, each follow-up visit within the first year after the initial follow-up, or until recurrence (whichever occurred first). This outcome was evaluated using the European Organisation for Research and Treatment of Cancer 30-item Core Quality-of-Life Questionnaire (EORTC QLQ-C30; score range: 0-100, with the highest score indicating optimal health or functioning), its 28-item ovarian cancer module (QLQ-OV28; score range: 0-100, with 100 indicating worst possible severity in the symptom scale and 100 indicating the best possible functioning or outcome in the functional scale), and Functional Assessment of Cancer Therapy-General (FACT-G7; score range: 0-28, with the highest score indicating best possible quality of life).

### Statistical Analysis 

Monthly accrual rates were estimated using locally weighted scatterplot smoothing to reduce noise inherent in monthly estimates. Crossover and complete cytoreduction rates were calculated for evaluable patients in each arm based on the assigned treatment, with sensitivity analysis evaluating the potential impact of missing data. Details on the sample size and power considerations are provided in the eMethods in Supplement 2. With a 1-sided, studywise, type I error level of .2, this lead-in study had 76.6% power to simultaneously reject the null hypotheses that (1) the patient accrual rate achieved in the final month of the lead-in phase would be 4.5 patients per month or fewer, (2) conversion to laparotomy would occur in 32% or more of patients in the MIS arm, and (3) the complete gross resection rate among the patients assigned to MIS would be no more than 27 percentage points lower than the rate of patients assigned to laparotomy.

We used medians (IQRs) to describe continuous variables and counts (proportions) for binary and categorical variables. We calculated median differences with 95% CIs obtained from quantile regression for continuous outcomes, which were not normally distributed, and relative risks (RRs) with Wald 95% CIs for binary outcomes. Differences in the HRQOL outcome were evaluated using mixed-effect linear regression models with random intercept terms; in these models, the assigned treatment was the exposure of interest, and the baseline survey score, age, weight, and marital status were included as covariates to increase precision. Race and ethnicity were self-reported by patients and were collected to assess potential disparities in treatment outcomes and ensure the findings are generalizable across diverse populations. We considered estimates of relative or absolute differences to be statistically significant when the 95% CIs excluded the null. All analyses were performed using Stata, version 16 (StataCorp LLC).

## Results

Of the 100 patients (median [IQR] age, 63 [39-82] years) included, 49 were assigned to MIS and 51 were assigned to laparotomy. One patient in the MIS arm became ineligible due to delay of surgery, and 4 patients in the laparotomy arm withdrew consent before surgery and were not evaluable for surgical outcomes. The patient flow diagram is shown in Figure 1, and baseline patient characteristics are shown in Table 1. Most patients identified as being White individuals (46 [90%] in the laparotomy arm, and 44 [90%] in the MIS arm) and not having Hispanic or Latino ethnicity (36 [71%] in the laparotomy arm, and 31 [63%] in the MIS arm). Most patients were diagnosed with stage IIIC cancer (34 [67%] in the laparotomy arm, and 33 [67%] in the MIS arm). Among the patients with known *BRCA* status at the time of randomization, 8 of 33 patients (24%) in the laparotomy arm and 9 of 34 patients (27%) in the MIS arm had a deleterious germline variation in the *BRCA1* or *BRCA2* gene.

**Table 1.** Patient Characteristics

Characteristic	Patients, No. (%)	
	Laparotomy arm (n = 51)	MIS arm (n = 49)
Age, median (IQR), y	63 (57-70)	61 (55-68)
Race^a^		
Asian	1 (2)	3 (6)
Black or African American	3 (6)	0
White	46 (90)	44 (90)
Other^b^	1 (2)	2 (4)
Ethnicity^a^		
Hispanic or Latino	15 (29)	18 (37)
Not Hispanic or Latino	36 (71)	31 (63)
Primary tumor		
Ovary	40 (78)	42 (86)
Fallopian tube	2 (4)	0
Peritoneum	9 (18)	7 (14)
Histologic type		
Serous	48 (94)	46 (94)
Other high-grade adenocarcinomas	3 (6)	1 (2)
Unknown	0	2 (4)
Preoperative *BRCA* status		
Wild type	25 (49)	25 (51)
*BRCA1* mutated	5 (10)	3 (6)
*BRCA2* mutated	3 (6)	6 (12)
Unknown	18 (35)	15 (30)
Cancer stage		
IIIC	34 (67)	33 (67)
IVA	8 (16)	9 (18)
IVB	9 (17)	7 (15)
MIS approach		
Traditional laparoscopy	NA	48 (98)
Robotic	NA	1 (2)
Planned HIPEC		
No	40 (78)	40 (82)
Yes	11 (22)	9 (18)
Surgical complexity score, median (IQR)	2 (1-3)	2 (1-3)

Abbreviations: HIPEC, hyperthermic intraperitoneal chemotherapy; MIS, minimally invasive surgery; NA, not applicable.

^a^ Race and ethnicity were self-reported by patients.

^b^ Other category included unknown.

**Figure 2.**
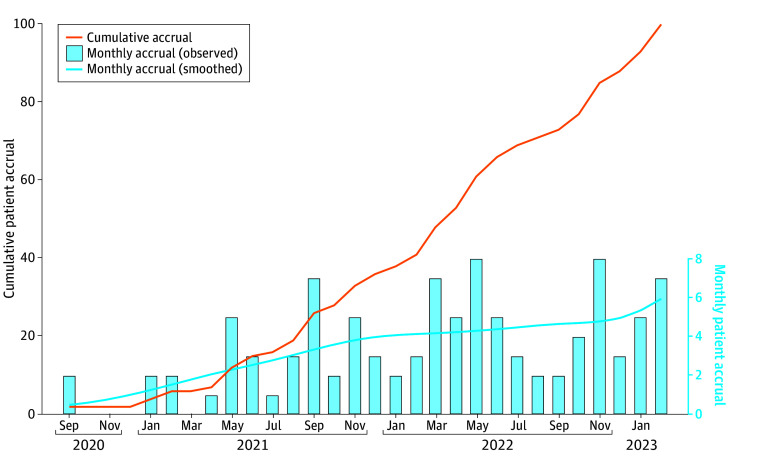
Cumulative and Monthly Accrual in the Lead-In Pilot Phase The cumulative accrual curve (solid line) depicts the total number of patients randomized by the end of each month. The bars indicate the number of patients randomized in each month, and the dashed line indicates estimated monthly accrual rate using locally weighted scatterplot smoothing. The monthly accrual rate reached 5.9 patients per month in February 2023, exceeding the prespecified feasibility threshold of 5.6 patients per month.

All 3 primary feasibility end points were met, indicating that the LANCE trial is feasible. In the last completed month of the lead-in pilot phase, the accrual rate reached 5.9 patients per month, exceeding the prespecified feasibility threshold of 5.6 patients per month (Figure 2). Six of 48 evaluable patients (12.5%; 95% CI, 4.7%- 25.2%) assigned to receive MIS underwent conversion to laparotomy. With these 6 conversions, there was inadequate exposure to assess the abdominal cavity in 2 patients, with 1 case due to substantial bowel adhesions. In 3 patients, cytoreduction was not deemed possible via laparoscopy due to tumor burden, with 1 case due to substantial bowel adhesions. In 1 patient, there was a combination of inadequate exposure to assess the abdominal cavity and the impossibility of cytoreduction via laparoscopy. To provide a conservative estimate of conversion rate, we assumed that the 1 unevaluable patient in the MIS arm would have had a conversion to laparotomy, which resulted in a conversion rate of 14.2% (95% CI, 5.9%-27.2%), which was below the prespecified threshold.

Complete gross resection was achieved in 42 of 48 (88%) evaluable patients who were assigned to MIS and 39 of 47 patients (83%) assigned to laparotomy, corresponding to an absolute difference of 4.5 (95% CI, −9.7 to 18.8) percentage points. To provide the most conservative estimate of the potential inferiority of MIS in achieving complete gross resection, the 4 unevaluable patients in the laparotomy arm were assumed to have had a complete gross resection, whereas the 1 unevaluable patient in the MIS arm was assumed to have had measurable residual disease. Under these assumptions, complete gross resection was achieved in 42 of 49 (86%) patients assigned to MIS and 43 of 51 (84%) assigned to laparotomy, corresponding to an absolute difference of 1.4 (95% CI, −12.6 to 15.4) percentage points.

Perioperative outcomes are provided in Table 2. Compared with the patients assigned to laparotomy, patients in the MIS arm had shorter median postoperative hospital stays (median [IQR], 1 [0-4] vs 4 [3-6] days; median difference, −3 [95% CI, −4 to −2] days) and lower estimated blood loss (median [IQR], 100 [50-250] mL vs 200 [100-400] mL; median difference, −100 [95% CI, −166 to −33] mL). There were 3 intraoperative complications in the MIS arm (1 diaphragmatic injury, 1 vascular injury, and 1 hemorrhage) and 3 in the laparotomy group (2 bladder injuries and 1 diaphragmatic injury), corresponding to an RR of any intraoperative complication of 0.98 (95% CI, 0.21-4.61). Four of 48 patients (8%) in the MIS arm had at least 1 postoperative complication, which was significantly fewer than the 12 of 47 patients (26%) in the laparotomy arm (RR, 0.33; 95% CI, 0.11-0.94). The differences in the complication rates were driven by CTCAE grade 1 to 3 complications (eTable in Supplement 2). Two patients in the MIS arm experienced a CTCAE grade 4 to 5 adverse event after surgery: 1 had reoperation to treat bowel ischemia, and 1 died of sepsis. There was 1 CTCAE grade 4 liver complication in the laparotomy arm.

**Table 2.** Perioperative Outcomes

Outcome	Patients, No. (%)		Median difference or RR (95% CI)^a^
	Laparotomy arm (n = 47)	MIS arm (n = 48)	
Length of stay, median (IQR), d	4 (3-6)	1 (0-4)	median: −3 (−4 to −2)
Estimated blood loss, median (IQR), mL	200 (100-400)	100 (50-250)	median: −100 (−166 to −33)
Any intraoperative complication	3 (6)	3 (6)	RR: 0.98 (0.21-4.61)
Any postoperative complication	12 (26)	4 (8)	RR: 0.33 (0.11-0.94)
Any gross residual disease	8 (17)	6 (13)	RR: 0.73 (0.28-1.95)

Abbreviations: MIS, minimally invasive surgery; NA, not applicable; RR, relative risk.

^a^ Median differences are for continuous and discrete variables; RRs are for binary variables.

**Figure 3.**
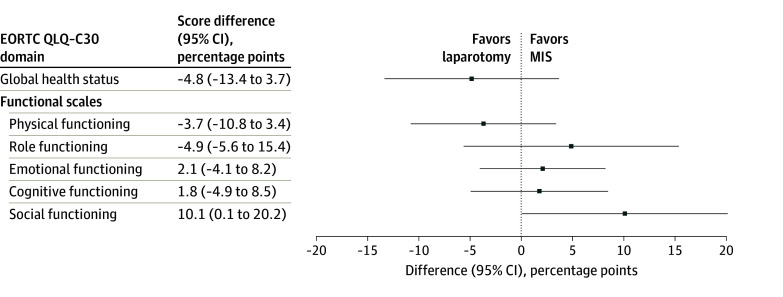
Health-Related Quality-of-Life Domain Score Differences Between Patients Randomized to Minimally Invasive Surgery (MIS) vs Laparotomy Questionnaires were administered at baseline and the first postoperative visit. Difference in scores after surgery (squares) and 95% CIs (horizonal bars) were estimated from random-effects models, adjusting for baseline scores, time since surgery, age, weight, marital status, and receipt of hyperthermic intraperitoneal chemotherapy. EORTC QLQ-C30 indicates European Organisation for Research and Treatment of Cancer 30-item Core Quality-of-Life Questionnaire.

At the first postoperative visit, which occurred a mean (SD) of 29.6 (11.0) days and 30.6 (12.1) days after surgery in the MIS and laparotomy arms, respectively (median difference, −1.0 [95% CI, −4.0 to 6.0] days), the global health status, as measured by the EORTC QLQ-C30, was similar for the patients assigned to MIS vs laparotomy (score difference, −4.8 [95% CI, −13.4 to 3.7] points). Among the functional scales, only the social functioning score was significantly higher in the MIS arm than in the laparotomy arm (score difference, 10.1 [95% CI, 0.1-20.2] points). Physical, role, emotional, and cognitive functioning scores did not differ significantly among the study groups (Figure 3). However, the CIs were wide and compatible with the presence of clinically important differences.

## Discussion

Results of this lead-in pilot phase of the LANCE trial showed that it is feasible to achieve an adequate patient accrual rate while maintaining a low rate of treatment crossover and achieving a comparable proportion of complete gross resection among patients randomly assigned to receive either MIS or laparotomy. Therefore, phase 3 of the LANCE trial, which will compare the oncological efficacy of MIS vs laparotomy, is feasible. Enrollment in this phase is ongoing.

Additionally, this pilot study provided evidence that, compared with laparotomy, MIS may reduce the risk of postoperative complications, which is consistent with findings in prior studies comparing MIS with open surgery in gynecologic oncology.^30,31^ The median duration of postoperative hospitalization was 3 days shorter after MIS vs after laparotomy. Furthermore, we found that social function, as measured with the EORTC QLQ-C30 at the first postoperative visit, was better among the patients assigned to MIS compared with those assigned to laparotomy. There were no measurable benefits in any other quality-of-life domain; however, there was substantial uncertainty in the estimates because of this trial’s small sample size, and it is possible that additional benefits of MIS will be noted in phase 3 of the LANCE RCT.

Studies have shown that compared with patients undergoing open abdominal surgery patients receiving MIS have a lower risk of postoperative complications, shorter hospital stays, faster recoveries, and reduced financial burdens.^17,32^ However, the potential benefits of MIS must be weighed against the risk of poor long-term oncological outcomes. Although findings from observational studies evaluating survival outcomes among patients who receive MIS for advanced-stage EOC after NACT are promising, it remains uncertain whether oncological outcomes after MIS are comparable with those achieved after laparotomy. Most observational studies have reported favorable outcomes—including high rates of complete cytoreduction, good perioperative outcomes, and excellent progression-free and overall survival—among women who underwent MIS cytoreduction after responding to NACT.^13,14,15,17,18,19,20^ However, an observational study of patients with advanced ovarian cancer reported nonsignificant decreases in cancer-specific survival and chemotherapy-free survival among women who had undergone interval cytoreductive surgery by MIS compared with those who had undergone laparotmy.^16^ Moreover, the premature and inappropriate adoption of MIS for the treatment of cervical cancer underscores the importance of RCTs of MIS for oncological surgery.^26,33^

### Strengths and Limitations

This trial has several strengths. To our knowledge, it was the first to examine MIS for interval cytoreduction of advanced ovarian cancer. Additionally, patients were enrolled from a diverse group of institutions in North America and Europe.

There are, however, limitations to this trial. First, it was designed to assess the feasibility of proceeding with phase 3 of the LANCE trial and was therefore not powered to detect differences in any of the secondary outcomes. For rare outcomes, such as intraoperative complications, the absence of a statistically significant difference does not necessarily imply the absence of a clinically important difference that might be detected after additional patient enrollment. Second, the participants had an exceptionally good response to NACT; thus, the findings may not be generalizable to patients whose CA-125 levels did not normalize after 3 to 4 cycles of NACT. To increase generalizability, phase 3 of the LANCE trial will include patients whose CA-125 levels do not normalize under laparoscopic assessment, demonstrating that complete cytoreduction is achievable with MIS. Third, we did not examine oncological outcomes in this study, and favorable findings regarding postoperative complications and improved social functioning cannot justify the routine use of MIS in the absence of high-quality evidence of the oncological efficacy of MIS.

## Conclusions

Results of this lead-in pilot phase of the LANCE trial demonstrated the feasibility of conducting phase 3 and highlighted some of the potential benefits of MIS. The complete LANCE trial may provide additional evidence of the efficacy of MIS and could facilitate informed decision-making in the treatment of advanced EOC after NACT.

## Notes

Supplement 1.
Trial Protocol

Supplement 2. **eMethods.** Power Calculations **eTable.** Postoperative Complications by Grade and Treatment
eReferences

Supplement 3.
Data Sharing Statement
