# Supplementary material for: Laparoscopic Cytoreduction After Neoadjuvant Chemotherapy in High-Grade Epithelial Ovarian Cancer: A LANCE Randomized Clinical Trial
Source: JAMA Netw Open. 2024 Nov 21;7(11):e2446325. doi: 10.1001/jamanetworkopen.2024.46325 (PMC11582931; doi:10.1001/jamanetworkopen.2024.46325)
Supplement: Supplement 1. — Trial Protocol [file jamanetwopen-e2446325-s001.pdf]

Laparoscopic cytoreduction After Neoadjuvant ChEmotherapy (LANCE)

MD Anderson Protocol Number: 2020-0165

Version Date: June 16, 2023

Coordinating Center

University of Texas MD Anderson Cancer Center
Department of Gynecologic Oncology & Reproductive Medicine
1155 Pressler St. Unit 1362
Houston, Texas, 77030

Principal Investigator

J. Alejandro Rauh-Hain, MD, MPH
Associate Professor
Department of Gynecologic Oncology & Reproductive Medicine
713-794-1759
jarauh@mdanderson.org

Co-Principal Investigator

Houston Methodist Hospital
Pedro T. Ramirez, MD
Chair
Department of Obstetrics & Gynecology
713-441-3193
pramirez3@houstonmethodist.org

Collaborators

Ying Yuan, PhD
Professor
Department of Biostatistics
713-563-4271
yyuan@mdanderson.org

Barrett C. Lawson, MD
Assistant Professor
Department of Pathology
713-563-9432
bclawson@mdanderson.org

Massachusetts General Hospital
Alexander Melamed, MD, MPH
Assistant in Gynecology and Obstetrics
617-724-4800
alexandermelamed@mgh.harvard.edu

Policlinico A. Gemelli Foundation,
Anna Fagotti, MD, PhD
Professor
Department of Woman and Child Health, Woman Health Area
011-39-339-379-9622
annafagotti70@gmail.com

TABLE OF CONTENTS

1. Background **3**
1.1 Epidemiology & Prognosis 3
1.2 Standard Therapies 3
1.3 State of the Art 4
1.4 Study Rationale 5
2. Study Objectives **5**
2.1 Primary 5
2.2 Secondary 5
3. Study Plan **5**
3.1 Study Design 5
3.2 Eligibility of Subjects 6
3.3 Screen & Baseline failures 6
3.4 Study Duration 7
3.5 Study Schema 7
4. Study Procedures **8**
4.1 Screening & Enrollment 8
4.2 Pre-operative Treatment & Assessments 8
4.3 Randomization 9
4.4 Surgical Procedures 9
4.5 Post-operative Procedures 11
4.6 Follow-up Phase (5 Years) 12
4.7 Recurrence/Progression 13
4.8 HR-QOL Questionnaires 13
4.9 Study Duration 14
4.10 Schedule of Assessments 15
5. Statistical Considerations **17**
5.1 Sample Size 17
5.2 Study Populations 17
5.3 Stopping Rules 17
5.4 Statistical Analysis 17
5.5 Interim Analysis 20
6. Quality Assurance **21**
7. Safety and Adverse Events **22**
7.1 Adverse Events 22
7.2 AE Description 24
7.3 Reporting of SAE 26
7.4 Death Notification 27
8. References **28**

1. BACKGROUND

1.1 EPIDEMIOLOGY & PROGNOSIS

Epithelial ovarian cancer (EOC) is the eighth most common cancer among women, and it represents about 4% of all women's cancers. According to global estimates, 225,000 new cases are detected each year, and 140,000 people annually die from the disease. Although ovarian cancer is only the eighth leading cause of cancer in women, it is the fifth leading cause of death [1-2]. Patients with EOC typically do not present with acute symptoms or dramatic findings on physical exam. Due to the lack of obvious symptoms and frequent diagnostic delays, EOC often goes undetected until it has spread within the pelvis and abdomen [3]. These tumors can be chemotherapy sensitive, enabling many patients to live for years with their disease. However, cure rates remain low. Five-year survival rates for women with advanced disease range from 20% to 40% [4-6].

1.2 STANDARD THERAPIES

EOC may be one of the only solid tumors where resection of metastatic disease is a standard part of initial management. Joseph V. Meigs, a gynecologic surgeon at Massachusetts General Hospital, initially described ovarian tumor debulking (cytoreduction) surgery in 1934. Over the following few decades, the concept did not gain wide acceptance, mainly due to a general lack of effective chemotherapeutic agents. Subsequently in the 1970s, C. Thomas Griffiths demonstrated that successful surgical cytoreduction (debulking) to a residual tumor size of ≤1.5 cm (now 1 cm) maximum diameter was associated with superior survival [7]. Thereafter many centers adopted increasingly aggressive surgical maneuvers aimed at resection of all visible disease prior to administration of chemotherapy [8-9].

Laparotomy is often both the diagnostic procedure and initial therapeutic intervention in advanced ovarian cancer. Surgical cytoreduction has several purposes: (1) it removes some or all of the tumor, (2) it improves physiology (GI obstruction and protein loss to ascites), (3) it removes de novo chemotherapy-resistant clones, and (4) it facilitates drug delivery by removing tumor with a compromised blood supply [10-11]. The operation at a minimum involves removal of the uterus, cervix, bilateral fallopian tubes and ovaries, and the omentum. Due to local cancer extension, other procedures, such as bowel surgery or removal of other organs in the abdomen are frequently required to remove all traces of tumor. Patients are either "optimally" (<1 cm residual disease) or "suboptimally" cytoreduced, a categorization that is the second only to stage in prognostic significance. More recent data suggest that cytoreduction to no gross residual disease should be the goal of cytoreductive surgery [12]. Patients with advanced stage disease require adjuvant chemotherapy consisting of carboplatin and paclitaxel with or without maintenance treatment with an angiogenesis inhibitor (i.e. bevacizumab) or a poly (ADP-ribose) polymerase (PARP) inhibitor.

Neoadjuvant chemotherapy (NACT) with interval cytoreductive surgery and post-operative chemotherapy is an alternative approach, which is often used to treat patients with large tumor burdens, multiple comorbidities, or stage IV disease [13-14]. Two phase III trials have found that women with advanced EOC who were randomized to NACT followed by interval debulking surgery have equivalent survival to those assigned to primary cytoreductive surgery and adjuvant chemotherapy [15-16]. A recent meta-analysis was consistent with these findings [17]. While primary cytoreductive surgery was the traditional treatment for advanced EOC, utilization of NACT has increased substantially in the United States [18]. By 2016, 1 in 3 patients with stage IIIC or IV epithelial ovarian cancer received neoadjuvant chemotherapy **(Figure 1)**. Recent guidelines from the American Society of Clinical Oncology and the Society of Gynecologic Oncology recommend NACT and interval surgery for women with high

perioperative risk, or low likelihood of achieving optimal cytoreduction at primary surgery [19]. In some women who have a response to NACT, interval cytoreduction can be achieved using minimally invasive surgery (MIS), but it remains unknown if MIS interval cytoreduction results in oncologic outcomes that are equivalent to those of interval cytoreduction completed via an open abdominal incision.

1.3 STATE OF THE ART

MIS has become common in gynecologic practice [19-24]. Previous studies that have investigated MIS in other anatomic sites have yielded contradictory results. For example, among women with early stage endometrial cancer, MIS improves perioperative outcomes without impairing survival [20-2] whereas in early-stage cervical cancer, MIS radical hysterectomy, until recently the standard approach, is inferior to open radical hysterectomy [22-23]. These conflicting outcomes underscore the importance of a randomized trial that will evaluate the oncologic efficacy of MIS interval debulking surgery. Moreover, despite the absence of high-quality evidence, the use of MIS for interval cytoreductive surgeries is on the rise **(Figure 1)**, reaching 21% in 2016.

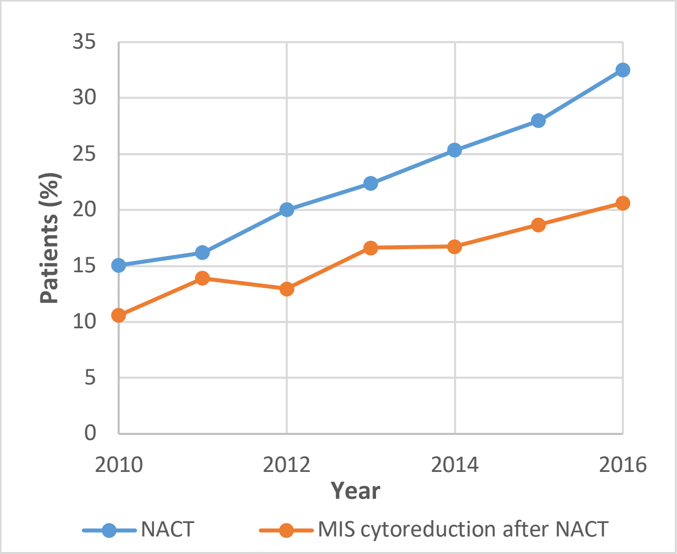

Figure 1. Percent of patients having MIS after NACT vs NACT alone

The proposed trial builds on observational studies [24-28] which have demonstrated a high rate of complete cytoreduction, good perioperative outcomes, and excellent progression-free survival among women who underwent MIS interval cytoreduction after responding to NACT. In a prospective observational study, Gueli Alletti et al. [26] performed MIS cytoreduction in 30 women with clinical response to NACT, and achieved resection of all visible disease in 29 women. All patients were alive with a median follow up of 10.5 months. Similarly, in a retrospective study of 30 women, Corrado et al. [26] found that interval MIS cytoreduction was associated with low rates of intra- and postoperative complication (3.3% and 6.6% respectively). At study completion, 26 of 30 patients were alive without recurrence with a median follow up of 15 months. In a historically controlled study of 10 women who underwent MIS interval cytoreduction, and 11 women who had laparotomy, Favero et al. [28] found a non-significant decrease in cancer-specific survival, and a non-significant reduction in the chemotherapy-free interval among women undergoing MIS. Melamed et al. [29] conducted a retrospective cohort study utilizing the National Cancer Database to evaluate the use and effectiveness of laparoscopic cytoreductive surgery among patients with advanced EOC that received NACT. These investigators compared 450 women who underwent MIS interval cytoreduction with 2,621 women who underwent laparotomy. Surgery was completed laparoscopically in 378 women (84% of cases initiated MIS) and there was no difference in overall survival or surgical outcomes between these groups, even after adjusting for numerous potential confounders. This study also suggested that a MIS approach might be reasonable and effective in well-selected patients. Importantly, there was a significant increase in the frequency of MIS interval cytoreduction in the United States from 2010 to 2012 (11–16%, $P<0.001$).

1.4 STUDY RATIONALE

At this time, available evidence suggests equipoise between MIS and open interval cytoreductive surgery for women with advanced EOC who have had a complete, or near complete, response to NACT. A randomized trial comparing these approaches is warranted. We propose an international validation study of interval MIS cytoreductive surgery. We will compare outcomes of patients that received NACT for advanced stage EOC who undergo a MIS procedure versus an open procedure.

2. STUDY OBJECTIVES

2.1 PRIMARY

The primary objective of this study is to examine whether MIS is non-inferior to laparotomy in terms of disease free survival (DFS) in women with advanced stage EOC that received 3 to 4 cycles of NACT.

2.2 SECONDARY

The secondary objectives of this study are as follows.

1. To determine if there are differences in Health-Related Quality of life (HR-QoL) in patients undergoing MIS vs laparotomy as assessed with the European Organisation for Research and Treatment of Cancer (EORTC) Quality of Life Questionnaire-Core 30 (QLQ-C30), QLQ-OV28, and Functional Assessment of Cancer Therapy-General (FACT-G7)
2. To determine if there are differences between patients undergoing MIS vs laparotomy in the rate of optimal cytoreduction (defined as residual tumor nodules each measuring 1 cm or less in maximum diameter) and complete cytoreduction (defined as no evidence of macroscopic disease)
3. To examine whether MIS is non-inferior to laparotomy in terms of overall survival (OS) in women with advanced stage EOC that received 3 to 4 cycles of NACT
4. To determine if there are differences between patients undergoing MIS vs laparotomy in surgical morbidity and mortality, intraoperative injuries, and post-operative complications
5. To determine the rates of MIS converted to laparotomy and the reasons
6. To determine if there are any difference in costs and cost-effectiveness between patients undergoing MIS vs laparotomy

3. STUDY PLAN

3.1 STUDY DESIGN

The study is an international, prospective, randomized, multicenter, non-inferiority phase III trial to compare MIS vs. laparotomy in terms of DFS in women with advanced stage high-grade EOC that had a complete or partial response to 3 or 4 cycles of NACT (see Study Schema in **Figure 2**).Up to 580 patients will be randomized prospectively prior to surgery (by minimization) in a 1:1 ratio to one of the 2 study arms:

- **Arm A** (experimental arm): patients will have MIS or
- **Arm B** (reference arm): patients will have a laparotomy.

The first 100 subjects will be enrolled into a pilot lead-in to determine feasibility based on criteria described in **Section 5.5.1**. If the study is determined to be feasible, all remaining subjects will be enrolled into the Phase III portion with interim analyses performed as described in **Section 5.5.2**.

3.2 ELIGIBILITY OF SUBJECTS

A subject will be considered **eligible** for inclusion in this study if all the following criteria are met.

1. Age ≥ 18 years old
2. Stage IIIC or IV, high-grade (serous, endometrioid, clear-cell, transitional carcinomas), invasive epithelial ovarian carcinoma, primary peritoneal carcinoma, or fallopian-tube carcinoma or pathology consistent with high-grade mullerian carcinoma.
3. Patient is considered by treating physician to be a surgical candidate after completion of 3 to 4 cycles of platinum-based chemotherapy, or an investigational neoadjuvant regimen given according to protocol, with complete radiologic resolution of any disease outside the abdominal cavity. Pleural effusions are acceptable per the local PI's discretion.
4. Normalization of CA-125 according to individual participating center reference range (Note: Among patients with a normal CA-125 at initiation of therapy, the CA-125 cannot exceed 35 U/mL at the completion of NACT prior to interval debulking surgery.) or has a CA-125 value ≤500 and is scheduled to undergo a diagnostic laparoscopy prior to debulking surgery.
    a. For patients undergoing diagnostic laparoscopy, surgeon considers that optimal debulking is feasible either by MIS or laparotomy.
5. Timeframe of ≤ 6 weeks (42 days) from the last cycle of NACT to interval debulking surgery. Overall timeframe may be extended per MD Anderson PI discretion.
6. ECOG performance status 0-2
7. Signed informed consent and ability to comply with follow-up
8. Negative pregnancy test by blood or urine (within 14 days prior to surgery)
9. Disease free of other active malignancies in the previous five years, except basal and squamous cell carcinomas of the skin

The subject must be **excluded** from participating in the study if any of the following criteria apply.

1. Evidence of tumor not amenable to minimally invasive resection on pre-operative imaging (CT, PET-CT, or MRI) including but not limited to the following findings that may preclude minimally invasive resection per surgeon's assessment.
    - Failure of improvement of ascites during NACT (trace ascites is allowed)
    - Small bowel or gastric tumor involvement
    - Colon or rectal tumor involvement
    - Diaphragmatic tumor involvement
    - Splenic or hepatic surface or parenchymal tumor involvement
    - Mesenteric tumor involvement
    - Tumor infiltration of the lesser peritoneal sac
2. History of psychological, familial, sociological or geographical condition potentially preventing compliance with the study protocol and follow-up schedule
3. Inability to tolerate prolonged Trendelenburg position or pneumoperitoneum as deemed by participating institution's clinicians
4. Any other contraindication to MIS as assessed by the clinician

3.3 SCREEN & BASELINE FAILURES

Study enrollment will occur at MD Anderson and participating sites. Participating site delegated staff will primarily screen for patients through the local site's EMR system. The LANCE study flier may be used for recruitment purposes if locally IRB approved. Written informed consent will be obtained via in person

or remote processes according to the local site's policies and standard operating procedures. A subject is considered to be a screen/baseline failure if the subject signs the informed consent but is determined to be ineligible or withdraws before study randomization. All potential subjects who are screened for enrollment in this study, including screening/baseline failures, will be listed on the Subject Screening Log/Identification List. Reasons for exclusion will be recorded for potential subjects who do not enter the study.

This is an internationally enrolling study. Informed consents and patient-facing materials have been and may be translated into the languages needed to ensure non-English speaking patients are accommodated to participate. Informed consent short forms or Verbal Translation Preparative Sheet for Non-English Speaking Subjects (VTPS) forms may be utilized if a consent translation is not available during the informed consent process.

3.4 STUDY DURATION

We anticipate a recruitment of up to 580 patients. Patients will be followed for a maximum of an additional 2 years after the final patient is enrolled or once the patient's 5 year Follow-Up Phase is complete, whichever occurs first.

3.5 STUDY SCHEMA

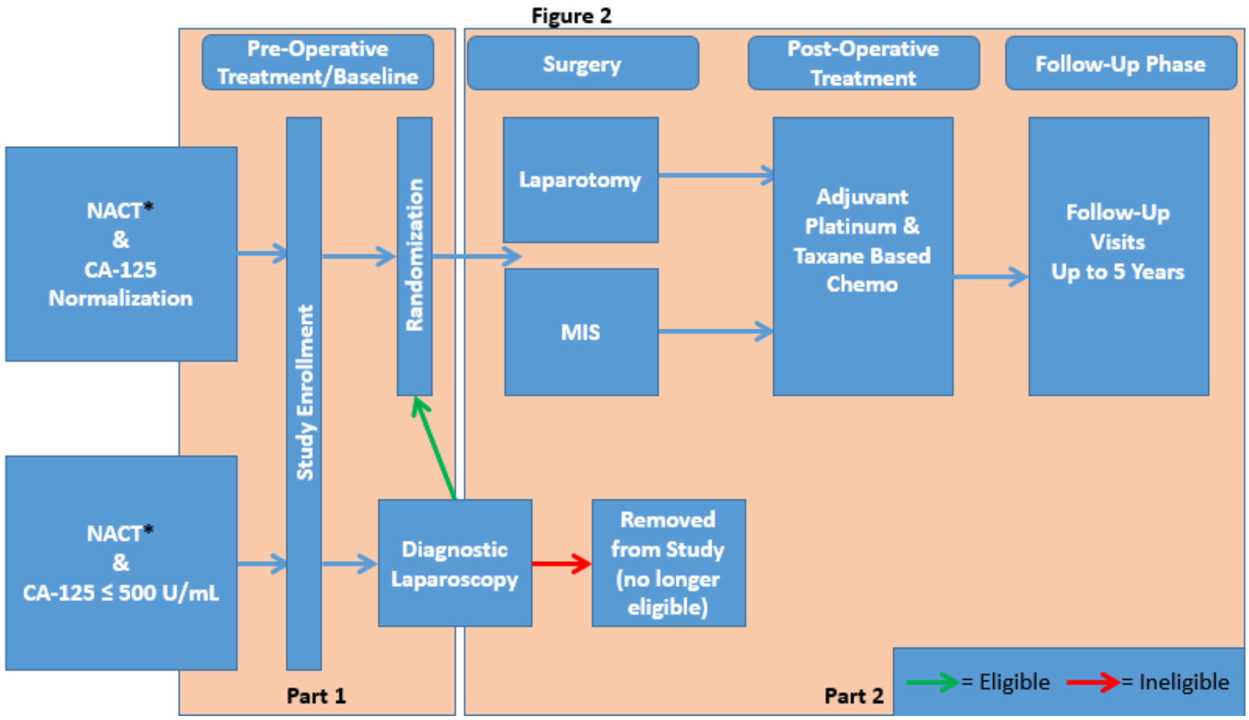

Figure 2. Study Schema

*Chemotherapy regimen will be documented before random assignment.

4. STUDY PROCEDURES

During the course of the study, all patients entering the study must be evaluated according to the Schedule of Assessments (**Table 1**).

4.1 SCREENING & ENROLLMENT

The following steps have to be completed within the 30 days prior to randomization:

- Informed consent (before performing any study-specific procedures) and
- Eligibility criteria verification.

A unique enrollment number will be allocated to each screened patient. This will be obtained through REDCap and is used to identify the patient on the electronic case report forms (eCRFs). If a patient withdraws from participation in the study, then her enrollment/randomization code cannot be reused.

4.2 PRE-OPERATIVE TREATMENT & ASSESSMENTS

The intended chemotherapy regimen will be established on an individual basis and will depend on patient's fitness, choice, and usual local practice, and will be pre-specified before random assignment. The recommended NACT regimen will be paclitaxel (175 mg/m^2 of body surface) administered as a 3-hour infusion immediately followed by an intravenous infusion of carboplatin (area under the curve of 6) over 1 hour. However, other regimens and combinations will be allowed, including but not limited to those in the following list:

- Carboplatin at an area under the curve (AUC) of 6 plus weekly paclitaxel at a dose of 80 mg/m^2 of body surface over a period of 1 hour on days 1, 8, and 15 of a 21-day cycle.
- Cisplatin in a dose of at least 75 mg/m^2 plus paclitaxel 135 mg/m^2 of body surface as a 24-hour continuous intravenous infusion every 3 weeks.
- Carboplatin at an AUC of 2 plus paclitaxel 60 mg/m^2 of body surface every week [4, 33-35].
- Other regimens including cisplatin at a dose of at least 75 mg/m^2 every 3 weeks, carboplatin at an AUC of at least 5 will be allowed [36].
- The addition of bevacizumab 15 mg/kg every three weeks to the NACT regimens prior to interval cytoreductive surgery will be allowed [37].

Dose reductions and modifications of chemotherapy and investigational agents will be based on institutional standards and toxicity side effects according to research protocols if patients are included in other therapeutic clinical trials.

The assessments to determine the patient's study eligibility must be done within 45 days prior to randomization and include the following.

- Informed Consent
- Medical history
- Demographic data
- Review of post NACT imaging to determine if patient is a surgical candidate
- Complete physical examination that includes the following.
    - ECOG performance status (PS)
    - BMI
    - Vital signs
    - Abdominal examination

  - Pelvic examination if performed

- Serum CA-125 levels from diagnosis and post-NACT are required, remaining collections during NACT phase to be recorded if available
- Laboratory analysis as indicated by treating physician
- Negative serum or urine pregnancy test for woman of childbearing potential [human chorionic gonadotropin (HCG) level of less than 5 mIU/mL
- Health Related Quality of Life (HR-QoL*) questionnaires (EORTC QLQ-C30, QLQ-OV28, and FACT-G7)
- NACT start date, end date and regimen

* HR-QoL questionnaires are not to be completed prior to obtaining informed consent.

4.3 RANDOMIZATION

After completion of pre-randomization assessments, patients who meet all eligibility requirements will be randomized 1:1 by minimization (Pocock-Simon randomization method) to the following arms:

- **Arm A** (experimental arm): patients will have MIS or
- **Arm B** (reference arm): patients will have a laparotomy.

Randomization will be stratified by the following criteria using CTC (Clinical Trial Conduct) system by Department of Biostatistics at MD Anderson Cancer Center based on simple list stratification:

- Stage of the disease (IIIC vs IV),
- BRCA status (positive *vs* negative *vs* unknown,
- Receipt of hyperthermic intraperitoneal chemotherapy (HIPEC) (yes vs no).

The CTC system is located https://biostatistics.mdanderson.org/ClinicalTrialConduct, which is housed on a secure server at MDACC and maintained by the MDACC Department of Biostatistics. Access to the website will be gained through usernames and passwords provided by the MDACC Department of Biostatistics to the clinical team. Training on the use of the CTC website to randomize patients ,on the study will be provided by the biostatistical collaborators. This is an open-label trial with stratified randomization. When a patient is enrolled on the study, the research nurses of the study will enter patient's information on medical record number and stratification factors. Through the web interface, after the randomization button is clicked, the result of the randomization will be displayed on the screen for users to view. All data on randomization will be stored in a secure SQL server database.

4.4 SURGICAL PROCEDURES

All intraoperative surgical adverse events will be recorded.

The addition of heated intraperitoneal chemotherapy (HIPEC) at the time of interval cytoreductive surgery will be allowed as long as the surgical team is able to perform this procedure with either MIS or open approach. Institutional HIPEC protocols will be reviewed prior to enrollment, and only protocols approved by the protocol principal investigator (PI) of the study will be allowed.

4.4.1 MIS

MIS cytoreduction will be performed as soon as possible after hematological recovery, but within 6

weeks after the final cycle of NACT (overall timeframe may be extended per MD Anderson PI discretion). A maximal effort to resect all gross visible tumor must be performed (complete cytoreduction). The specifics of each operation will be at the discretion of the operating surgeon (e.g. port-site placement, omentectomy, etc.), as will the decision to convert to an open surgery. If the surgeon's judgement is that complete gross resection can only be accomplished by performing an open procedure, then the surgeon should convert to open surgery. Details relating to the planned and actual operation will be collected on the baseline and operative case report forms.

The procedures listed below are recommended but not required. If performed, documentation on the surgical report is required.

- Intra-abdominal access will be obtained (laparoscopy or robotic assisted laparoscopy).
- After confirming successful and safe entry into the abdominal cavity, careful exploration of the peritoneal cavity will be the next surgical step.
- In patients eligible for the MIS approach, three or four 5-mm and/or 10-mm trocars will be placed in standard position for pelvic surgery, robotic platforms will be used in standard surgical fashion. Additional ports may be added if required.
- Cytoreduction will consist of total/radical hysterectomy, bilateral salpingo-oophorectomy (BSO), omentectomy (infracolic omentectomy is allowed if there is no evidence of tumor in the omentum), pelvic, or upper peritonectomy.
- Pelvic and aortic lymphadenectomy will not be performed unless lymph nodes are abnormally enlarged.
- Additional procedures (e.g., anterior rectal resection or splenectomy) will be performed if needed.
- In cases of increased surgical complexity additional trocars may be placed.
- The use of a "mini-laparotomy," a small abdominal incision (≤ 8 cm), to facilitate the surgical procedure or to facilitate specimen extraction is permissible and not defined as a conversion to open laparotomy. If a mini-laparotomy is performed, the length of the incision must to be documented in the operative note with digital photograph confirmation of the size of the incision.
- In cases where optimal cytoreduction is not deemed possible via laparoscopy, the surgeon may convert to a laparotomy if optimal cytoreduction is felt to be feasible through an open approach. Conversion to laparotomy may also occur for other indications at the discretion of the surgical team (e.g. injury, exposure, intolerance of pneumoperitoneum, etc). Any incision larger than 8 cm will be categorized as a conversion to laparotomy. Prospectively completed forms will document reasons for conversion from laparoscopy to laparotomy and time of conversion.
- At the end of surgery, the largest diameter of any grossly visible residual tumor will be documented.

4.4.2 LAPAROTOMY

Laparotomy will be performed as soon as possible after hematological recovery, but within 6 weeks after the final cycle of NACT (overall timeframe may be extended per MD Anderson PI discretion). The procedures listed below are recommended but not required. If performed, documentation on the surgical report is required.

- Surgical access will be obtained via a midline (or paramedian) laparotomy incision.
- Careful exploration of the peritoneal cavity will be the next surgical step.
- Cytoreduction will consist of total/radical hysterectomy, BSO, omentectomy (infracolic omentectomy is allowed if there is no evidence of tumor in the omentum), pelvic, or upper peritonectomy.

- Pelvic and aortic lymphadenectomy will not be performed unless lymph nodes are abnormally enlarged.
- Additional procedures (e.g., anterior rectal resection, splenectomy, etc.) will be performed if needed.
- At the end of surgery, the largest diameter of any grossly visible residual tumor.

4.5 POST-OPERATIVE PROCEDURES

4.5.1 POST-OPERATIVE VISIT

A post-operative follow-up visit will be performed in person or by telemedicine 30 days (± 15 days) after surgery. Each participating site will follow their institutional guidelines for remote procedures.

The following assessments will be completed (unless noted as recommended but not required). All potential post-operative complications will be documented at the time of postoperative visit.

- A postoperative imaging study is recommended but not required (CT of the abdomen and pelvis with and without contrast both IV and oral within 30 days after surgery
- Complete physical examination that includes the following (when available):
  - ECOG PS
  - BMI
  - Vital signs
  - Abdominal examination
  - Pelvic examination if performed
- Laboratory analysis as indicated by primary clinician,
- Serum CA-125 levels, if available
- Health Related Quality of Life questionnaires (EORTC QLQ-C30, QLQ-OV28, and FACT-G7), and
- Post-operative AE (up to 30 days post-surgery and up to 6 months for adverse events of special interest [AESI]) and particularly:
  - Surgical morbidity (CTCAE v5.0)
  - Vital/Disease Status

4.5.2 POST-OPERATIVE TREATMENT

Post-operative treatment can be platinum and taxane based chemotherapy or other first-line FDA approved treatment or clinical trials, as described in the following two subsections.

4.5.2.1 PLATINUM AND TAXANE BASED CHEMOTHERAPY

Per physician discretion, post-operative chemotherapy may consist of completion of at least six cycles of platinum and taxane based chemotherapy. The first cycle of chemotherapy after surgery will be administered as deemed safe after surgery by the treating oncologist, but preferably no more than six weeks later.

The recommended post-operative regimen includes paclitaxel (175 mg/m^2 of body surface) administered as a 3-hour infusion immediately followed by an intravenous infusion of carboplatin (AUC of 6) over 1 hour. Other regimens include but are not limited to at least 75 mg/m^2 cisplatin every 3 weeks, carboplatin at an AUC of at least 5, or weekly 80 mg/m^2 body surface paclitaxel for 1 hour on days 1, 8, and 15 of a 21-day cycle. The addition of bevacizumab 15 mg/kg every three weeks for a total of 22 cycles or bevacizumab 7.5 mg/kg every three weeks for a total of 18 cycles after interval cytoreductive surgery is allowed [38-39]. The addition of maintenance PARP inhibitors is allowed per physician discretion [40].

4.5.2.2 ALTERNATIVE POST-OPERATIVE, FIRST-LINE TREATMENT

Any future FDA approvals in the first line setting will be allowed.

4.6 FOLLOW-UP PHASE (5 YEARS)

Follow-up visits will occur for up to 5 years or until recurrence, whichever occurs first, at the following intervals:

- Every 3 months during the first two years (± 30 days) and then
- Every 6 months during the third-fifth year (± 30 days).

The end of adjuvant treatment visit marks the commencement of the Follow-Up Phase. The following assessments will be completed at the first Follow-Up Phase visit.

- Follow up imaging when available to assess for the presence of new disease
- Complete physical examination (when available), including:
    - ECOG PS
    - BMI
    - Vital signs
    - Abdominal examination
    - Pelvic examination, if performed
- CA-125 serum levels, if available
- Laboratory analysis as indicated by primary clinician
- Health Related Quality of Life questionnaires* (EORTC QLQ-C30, QLQ-OV28, and FACT-G7)
- Chemotherapy start date, end date and regimen will be documented at start of Follow-Up Phase (end of adjuvant treatment visit)
- Maintenance start date, end date and regimen will be documented at start of Follow Up Phase
- Genetic testing will be documented when available and/or sharable:
    - HRD status
    - BRCA germline/somatic status
- Vital/Disease Status

The following assessments will be completed at subsequent visits until recurrence.

- Imaging assessments to include pelvic, abdominal, and chest CT, PET/CT, or MRI in cases of rising CA-125 or symptoms concerning for recurrence including, but not limited to abdominal pain, bloating, anorexia, and unexplained nausea/vomiting
- Complete physical examination (when available), including:
    - ECOG PS
    - BMI
    - Vital signs
    - Abdominal examination
    - Pelvic examination, if performed
- CA-125 serum levels, if available
- Maintenance start date, end date and regimen will be documented at start of Follow Up Phase
- Health Related Quality of Life questionnaires* (EORTC QLQ-C30, QLQ-OV28, and FACT-G7)
- Vital/Disease Status

*See **Section 4.8.1** or **Table 1** for duration of HR-QoL administration.

4.7 RECURRENCE/PROGRESSION

Recurrence/progression will be determined primarily by the treating oncologist. In situations in which relapse status is unclear, a third party committee of gynecologic oncologists will review and document whether recurrence is present or not. Clinical Recurrence is defined as any of the following.

1. Radiologic recurrence as diagnosed by clinical radiologist using RECIST v1.1 as guidance, which defines recurrence as:
   - A new lesion or
   - 20% increase in sum of target lesions compared to smallest sum on study when increase is at least 5 mm (**Note:** Date of recurrence should be documented as the date new lesion or increase was observed).
2. CA-125 recurrence* defined using Gynecological Cancer Intergroup (GCIG) criteria as listed below.
   - For patients with elevated CA-125 pre-treatment and normalization at the completion of first line chemotherapy and patients with CA-125 in normal range before treatment: **CA-125 ≥ the upper limit of the reference range on two occasions** (**Note:** Date of recurrence should be documented as the date of the first CA-125 elevation ≥ the upper limit of the reference range)
   - For patients with elevated CA-125 pre-treatment, which never normalized: **CA-125 ≥ the nadir value on two occasions** (**Note:** Date of recurrence should be documented as the date of the first CA-125 elevation ≥ nadir value)

   * CA-125 recurrence defined using GCIG criteria without other evidence of recurrence based on imaging or pathologic confirmation should be discussed with the protocol PI prior to documenting as a recurrence.
3. Clinical progression as diagnosed by the treating physician is defined as overall deterioration of health.
4. Pathologic progression is defined as pathologic confirmation of new macroscopic tumor.

The date of progression for patients who are diagnosed with progression by more than one definition (for example both radiologic and pathologic) is defined as the date of the earliest type of progression event.

4.8 HR-QOL QUESTIONNAIRES

4.8.1 ADMINISTRATION

Paper or electronic questionnaires will be given to the patient at baseline (after obtaining consent and within 30 days prior to surgery) and then at every follow-up visit within the first year after end of treatment until recurrence, according to the Schedule of Assessments (**Table 1**). Patient is to complete the questionnaires during the clinic visit, electronically, or by phone based on **Table 1** timelines. If questionnaires are completed using paper, the site staff will enter the information directly into the study database.

Each center must allocate the responsibility for questionnaire administration to a specific person (e.g. a research nurse or study coordinator) and, if possible, assign a back-up person to cover if that person is absent. The significance and relevance of the data need to be explained carefully to participating patients so that they are motivated to comply with data collection.

The instructions for completion of the HR-QoL questionnaires are as follows.

- The patient must complete the questionnaire in private.

- The patient should be given sufficient time to complete at their own speed.
- The patient should not receive help from relatives, friends or clinic staff to answer the questionnaire. However, if the patient is unable to read the questionnaire (e.g., is blind or illiterate) the questionnaire may be read out by trained clinic staff and responses recorded.
- Only 1 answer should be recorded for each question.
- Upon completion of the questionnaire, it should be returned to the person responsible for questionnaires who should check for completeness.
- Missing or missed survey timepoints are not considered a deviation.

4.8.2 MEASURES

1. The EORTC QLQ-C30 is an integrated system for assessing the health-related quality of life of cancer patients participating in international clinical studies. The QLQ-C30 includes 30 items and measures five functional scales (physical, role, emotional, cognitive and social functioning), global health status (GHS), financial difficulties and eight symptom scales (fatigue, nausea and vomiting, pain, dyspnea, insomnia, appetite loss, constipation, diarrhea) [41]. These scores vary from 0 (worst) to 100 (best) for the functional dimensions and GHS, and from 0 (best) to 100 (worst) for the symptom dimensions and were generated according to the EORTC Scoring Manual [42].
2. The QLQ-OV28 is a supplemental module that contains question that are specifically relevant to the quality of life of patients with ovarian cancer. The questionnaire contains 28 that address seven scales including abdominal symptoms, peripheral neuropathy, chemotherapy side-effects, hormonal symptoms, body image, attitude to disease and treatment, and sexual functioning. These symptom domains are scored form 0 (best) to 100 (worst).
3. The FACT-G7 (Version 4) is a patient-reported outcome measure used to assess health-related quality of life in patients undergoing cancer therapy. This questionnaire is an abbreviated version that ask 7 questions from the original FACT-G instrument. These questions focus on domains that cancer patients report to be the most burdensome. This brief questionnaire is intended for routine clinical use and will be validate in this study.

4.9 STUDY DURATION

Subjects will be encouraged to complete the study activities and follow-up visits; however, they may voluntarily withdraw at any time. The investigator may also withdraw the subject from participating in this study at any time, or the protocol PI may discontinue the study.

Reasons for early withdrawal from the study should be documented in the CRF as follows:

- Study closed/terminated,
- Subject lost to follow-up within 30 days of enrollment,
- Investigator's decision, or
- Subject withdrew consent.

Withdrawal date and rationale will be documented in the subject's medical record and in the CRF. In the case of death, a death certificate should be obtained if possible. Cause of death should be evaluated and documented in the CRF.

Per institutional guidelines, patients that are taken off study may be approached for permission to continue data collection that becomes available in the EMR.

4.10 SCHEDULE OF ASSESSMENTS

Table 1 Schedule of Assessments

Time Point (TP)	Baseline	MIS or Laparotomy Surgery	Post-Operative Visit	Follow-Up Phase[a]		
**Visit Window**	**Within 30 Days from Randomization**	**N/A**	**30 Days (± 15 Days)**	**First Follow-Up Phase Visit**	**Year 1-2 Every 3 Months (± 30 Days)**	**Year 3-5 Every 6 Months (± 30 Days)**
**Informed Consent**	X					
**Medical History**	X					
**Demographic Data**	X					
**Tumor[b] Assessment**	X		X[c]	X[d]	X[e]	X[e]
**Physical Exam[f]**	X		X	X	X	X
**Serum CA-125**	X[g]		X[h]	X[h]	X[h]	X[h]
**Laboratory Analysis[h]**	X[i]		X[i]	X[i]		
**Pregnancy Test[j]**	X					
**HR-QoL[k]**	X		X	X	X[l]	
**BRCA & HRD Status**				X[m]		
**Adverse Events[n]**		X	X	X	X	
**Surgical Procedures[o]**		X				
**Anticancer Therapy Documentation**	X[p]			X[q]		

---

[a] End of adjuvant treatment visit marks the commencement of the Follow-Up Phase. Data will be collected until disease recurrence, then only followed for vital status and cause of death.

[b] To include pelvic, abdominal, and chest CT, PET/CT or MRI (only required at baseline) with RECIST guidance.

[c] CT of the abdomen and pelvis with and without contrast both IV and oral within 30 days after surgery is recommended but not required to document radiologic evidence of residual disease.

[d] Recommended but not required at end of treatment (not considering maintenance) to document radiologic evidence of residual disease.

[e] In the case of rising CA-125 or symptoms concerning for recurrence including, but not limited to: abdominal pain, bloating, anorexia, unexplained nausea/vomiting.

[f] To include ECOG PS, BMI, vital signs, abdominal exam, and pelvic exam if performed.

[g] To be recorded from diagnosis and post-NACT (required), remaining collections during NACT phase to be recorded if available.

[h] To be recorded if available.

[i] To include hemoglobin, creatinine, albumin.

[j] Negative serum or urine pregnancy test for woman of childbearing potential [human chorionic gonadotropin (HCG) level of less than 5 mIU/mL.

[k] EORTC QLQ-C30, QLQ-OV28, and FACT-G7. To be completed by the patient +/- 7 days from each actual clinic visit; if patient misses visit the original timeframes for collecting should be followed. Missing or missed survey timepoints are not considered a deviation.

[l] To be collected at every follow-up visit within the first year after First Follow-up Visit or until recurrence.

[m] To be documented when available and/or shareable at start of Follow-Up Phase and during surveillance.

[n] To be collected up to 30 days post-surgery and up to 6 months for AEs of interest: Surgical morbidity (CTCAE v5.0) and mortality. All potential adverse events complications will be documented at the time of the next study visit.

[o] See Protocol Section 4.4 (Surgical Procedures). Surgery to be completed within 6 weeks after last cycle of neoadjuvant chemotherapy.

[p] NACT start date, end date and regimen will be documented.

[q] Post-operative chemotherapy start date, end date, and regimen will be documented at start of Follow-Up Phase (end of adjuvant treatment visit). Maintenance treatments will also be documented at start of Follow-Up Phase and during surveillance.

5. STATISTICAL CONSIDERATIONS

5.1 SAMPLE SIZE

We aim to demonstrate a non-inferiority of MIS vs. laparotomy with a non-inferiority margin of 33% in Hazard Ratio (HR = 1.33). Under the assumptions of a median DFS of 23 months in both laparotomy and MIS arms, an accrual rate of 7 patients per month in the first 14 months after all pilot sites are open, and 20 patient per subsequent months after all sites open, a maximum of 549 patients (or 323 events) are required to yield 80% power, given a one-sided type I error rate of 5%. Taking into account 5% inevaluables/screen failures, a total of 580 patients will be enrolled across all participating sites.

5.2 STUDY POPULATIONS

All efficacy analyses will be conducted on the intention to treat (ITT) population. For non-inferiority objective analyses, we will perform a sensitivity analyses amongst the per-protocol (PP) population.

The ITT population will consist of all randomized subjects regardless of what treatment was actually received.

The PP population will consist of all subjects with at least one post baseline assessment follow for recurrence. Patients will be included in the treatment group received regardless of randomized assignment.

Safety analyses will be conducted on all randomized patients and with at least one post-baseline safety assessment.

5.3 STOPPING RULES

In addition to the planned interim analysis described in **Section 5.5**, the protocol PI reserves the right to terminate the study at any time. In terminating the study, the protocol PI will ensure that adequate consideration is given to the protection of the patient's interests.

The PI in each participating center needs to be notified about the end of the study or early termination of the study.

Consideration to stopping the study after 100 patients have been accrued will be given if any or at least one of the following events occur.

- Annual patient accrual is less than 30 for all sites.
- Less than 75% of patients are available for follow-up.
- There is an unacceptable rate of severe surgical related incidence of complications (>8%) in the MIS group (severe will be defined by CTCAE complications graded ≥4).

5.4 STATISTICAL ANALYSIS

A final statistical plan will be written before data is frozen. Statistical analyses will be performed using CRF data collected until a clinical cut-off date that is defined in function of the time when the number of events required for the interim and final analysis of the efficacy variables will be reported.

The ITT population will be used for the analyses of all efficacy endpoints, and PP analyses will be done as sensitivity analyses.

The ITT population will be used for QoL analyses.

The safety population will be used for reporting the safety data and treatment exposure data.

Subgroup analyses will be performed for selected efficacy endpoints.

Unless otherwise indicated in the analyses, we will present data by the two treatment arms.

All tests will be performed at a two-sided significance level of 5% (95% Confidence interval), with the exception of the tests for the primary endpoint of non-inferiority using one-sided test at a significance level of 2.5%.

The following conversion factors will be used to convert days into months or years: 1 month = 30.4375 days, 1 year = 365.25 days.

5.4.1 PRIMARY ENDPOINT

The primary objective of this prospective, multicenter, randomized, non-inferiority phase III study will be Disease Free Survival (DFS) (non-inferiority of MIS vs. laparotomy) in women with advanced stage EOC that received 3 to 4 cycles of NACT. The main analysis will be performed according the intention-to-treat (ITT) principle. DFS is defined as the time interval between randomization and physical or radiographic evidence of recurrence (local/distant) or death (all causes) whichever occur first. The onset of clinical progression is defined as either radiographic evidence of increasing disease as assessed by a clinical radiologist with (Response Evaluation Criteria in Solid Tumors (RECIST) criteria, version 1.1, [29] as guidance), an overall deterioration in health, or a rise in the CA-125 level as assessed with the use of the Gynecologic Cancer Inter Group criteria [31]. Kaplan Meier curves will be used to describe DFS over time. Log-rank test will be used to compare DFS between the control and experimental arms. The treatment effects will be summarized by means of a hazard ratio with its associated 95% confidence interval. Two years DFS rate will be computed with a targeted 95% Confidence Interval (CI). Median event-free times by treatment arm will be reported with 95% CI, if the number of events allows the estimation of the median. The CI for the median survival time will be calculated according to Brookmeyer, R. and Crowley, J. (1982). Event rates at specified time points will be estimated from the Kaplan-Meier curve. The standard error will be estimated by the Greenwood formula and the log-log transformation will be used to compute confidence intervals. When appropriate, multivariate Cox analyses will be performed, in which a univariate selection procedure will serve to identify eligible explanatory variables with univariate Cox (using Wald Test) p-value lower than 0.10 as potential prognostic value.

5.4.2 SECONDARY ENDPOINTS

Table 2. Outcome Measures and Populations for the Secondary Objectives

**Secondary Objective**	**Outcome measure**	**Population**
HR-QoL	HR-QoL of patients assessed with European Organisation for Research and Treatment of Cancer (EORTC) Quality of Life Questionnaire-Core 30 (QLQ-C30), QLQ-OV28 (ovarian supplement) and Functional Assessment of Cancer Therapy-General short-form (FACT-G7). Completed at study entry, within 30 days after surgery, and at each follow up visit.	mITT (patients with at least baseline HR-QoL score)
Optimal cytoreduction	Defined as residual tumor nodules each measuring 1 cm or less in maximum diameter.	ITT
Complete cytoreduction	Defined as no evidence of macroscopic disease.	ITT

Overall survival (OS)	Overall survival (OS) defined as time interval between randomization and death (all causes); living patients will be censored at the last date of news.	ITT
Surgical morbidity and mortality	Surgical morbidity (CTCAE v5.0 and mortality (30-day post-operative for adverse events and up to 6 months post-operative for adverse events of interest).	ITT
Intraoperative injuries	Coded as yes or no and categorized as involving the bowel, veins, arteries, ureter, bladder, or other site.	As treated
MIS converted to laparotomy	Prospectively completed forms documented reasons for conversion of MIS to laparotomy	As treated
A cost analysis may be performed in some countries	Cost of the procedure (option for some countries).	ITT

Depending on the data type of secondary endpoints, the following analytic approach will be used.

1. **Continuous Variables**

   Continuous variables will be summarized using descriptive statistics, i.e. number of patients with available data (N), mean, median, standard deviation (S.D.), 25% - 75% quartile (Q1-Q3), minimum, and maximum.

   Continuous variables could be transformed to categorical variables using the median or using conventional cut-offs from bibliography or clinical practice. Two-sample t-tests will be used to compare experimental and control arms. Linear regression will be used to compare the two arms while adjusting for potential confounding factors.

2. Categorical Variables

   Frequencies in tables will be presented by arm and total frequency and percentages and missing modality. Qualitative variables will be summarized by means of counts and percentages. Unless otherwise stated, the calculation of proportions will be based on the sample size of the population of interest. Chi-square test will be used to compare experimental and control arms. Logistic regression will be used to compare two arms while adjusting for potential confounding factors.

3. **Survival**

   Overall survival will be estimated using the Kaplan-Meier method, and will be described using the median with its 95% CI. Univariate Cox proportional hazards model (i.e., logrank test) will be used to estimate hazard ratios (HR: control arm versus investigational arm) with a 95% CI. When appropriate, multivariate Cox analyses will be performed, in which a univariate selection procedure will serve to identify eligible explanatory variables with univariate Cox (using Wald Test) p-value lower than 0.10 as potential prognostic value. Follow-up will be estimated using the reverse Kaplan-Meier method, and will be described using the median with its 95% CI.

4. **Safety**

   Safety data will be summarized using descriptive statistics, including mean, standard deviation and 95% confidence interval. AE and Serious AE (SAE) will be tabulated by type and severity. Fisher’s exact test or Chi-square tests will be used to compare two arms.

5. **HR-QoL**

   Post-surgery HR-QoL scores between the two randomized will be evaluated at 1 and 3 months

after surgery. A linear mixed model will be fitted for each HR-QoL scale score, adjusted for corresponding baseline assessment scores, time effect, patients' age, weight (in kilograms) at study entry, and marital status (married vs. not married). The interaction between treatment and time will be examined for the similarity of treatment effects at 1, and 3 months post-surgery, with the significance level for an interaction set at .05. If the interaction effect is significant, then treatment effects at each postsurgical assessment will be tested at the significance level of .025 by comparing least-square means between the two arms. Otherwise, the mixed model will be refitted without the interaction, and treatment effects will be tested as an overall effect over the postsurgical assessments. As an exploratory analysis a general linear model will be used to explore whether any difference persists at 6 months, adjusting for corresponding baseline scores, patients' age, weight at baseline, and marital status.

5.5 INTERIM ANALYSIS

5.5.1 PILOT LEAD-IN

The first 100 patients enrolled in this study will be enrolled in a lead-in pilot to assess the feasibility proceeding with the phase III portion of the study. The study will be considered feasible and will continue to Phase III under the following conditions: 1) the accrual rate reaches at least 80% of the target rate after all pilot sites are open (i.e., 7 patient per month), 2) the crossover rate in the MIS group is less than 25%, and 3) the difference of complete gross resection between the MIS and open group is less than 20%. Monthly recruitment rates will be calculated as (patients enrolled/months) and plotted over time. To reduce the effect of random variation, a locally weighted polynomial regression (LOWESS) will be used to calculate a smoothed enrollment rate versus time curve. The enrollment endpoint will be assessed using enrollment rate achieved by the last month of the study as predicted by the LOWESS curve. The co-primary crossover rate endpoint will be evaluated concurrently with the enrollment endpoint, and will be met if fewer than 25% of patients assigned to minimally invasive surgery undergo conversion to laparotomy.

If the feasibility endpoints are not reached, accrual will be suspended and consideration will be given to changing and/or adding sites, changing enrollment criteria, or terminating the trial. No formal futility/efficacy stopping monitoring will be performed in the first interim analysis. The feasibility results of the pilot lead-in and secondary endpoints (HR-QoL, residual disease status, AEs, safety, etc) will be reported regardless of the feasibility findings.

5.5.2 PHASE III

If the study proceeds to phase III enrollment, an interim analysis will occur when a total of 152 (i.e., 50% of expected) events (i.e., recurrence or second cancer or death) are observed in the two arms. A non-binding futility monitoring will be performed based on Lan-DeMets beta spending function, with stopping boundary of p value = 0.37. If the HR is ≥ 1.33 at interim analysis the study will be discontinued for safety concerns. At the end of the trial, the logrank test will be performed at a one-sided significance level of 0.05. The boundaries defined by the interim p-values for efficacy will be used as guidelines by Data and Safety Monitoring Board (DSMB)[1] along with all other relevant study information, including the safety data, to decide if it is appropriate to make a recommendation other than to continue the study as planned.

5.5.3 EARLY STUDY TERMINATION

[1] We will use the MD Anderson DMSB. The DSMB will have responsibility for monitoring, oversight of adverse events, and other protocol events

There is some concern as to whether there may be an increased rate of severe adverse events in the MIS group. The rate of surgery related death will be formally assessed after 50, 100, 150, 200, and 300 MIS patients have completed surgery. Early termination will be considered if there are surgery related deaths. The upper bounds of the number of deaths caused by surgery are presented in **Table 3**.

Table 3. Upper bounds for early study termination

Number of subjects in the MIS group	Consider stopping the study if the number of deaths caused by surgery is ≥
50	2
100	3
150	4
200	5
300	6

All toxicity data will be reviewed so that recommendations can be made in regards to changes to the protocol and/or stopping the trial. Additional revised power calculations will be undertaken at these times based on both local and total recurrence which may indicate that larger (or smaller) relapse rates may be worth detecting.

Toxicities/complications that will be considered include the following.

- Conversion to laparotomy as a result of bleeding
- Injury to abdominal or pelvic viscera secondary to endoscopic instrument placement or manipulation, including injury to the bowel, bladder, vessels, ureter or kidney
- Nerve injury requiring physical therapy or restricting function
- Unplanned ICU admission
- Death within 30 days of surgery

6. QUALITY ASSURANCE

The Data Quality Management Plan (DQMP) outlines the procedures required for institutions who desire to collaborate with the Department of Gynecologic Oncology & Reproductive Medicine at MD Anderson Cancer Center (Coordinating Center) in the conduct of this multicenter study. Please refer to Appendix A (DQMP).

Study data will be collected and managed using REDCap (Research Electronic Data Capture) electronic data capture tools hosted at MD Anderson. REDCap (www.project-redcap.org) is a secure, web-based application with controlled access designed to support data capture for research studies, providing: 1) an intuitive interface for validated data entry; 2) audit trails for tracking data manipulation and export procedures; 3) automated export procedures for seamless downloads to common statistical packages; and 4) procedures for importing data from external sources. In the case of multi-center studies REDCap uses Data Access Groups (DAGs) to ensure that personnel at each institution are blinded to the data from other institutions.

REDCap (https://redcap.mdanderson.org) is hosted on a secure server by MD Anderson Cancer Center's Department of Research Information Systems & Technology Services. REDCap has undergone a Governance Risk & Compliance Assessment (May 2014) by MD Anderson's Information Security Office and found to be compliant with HIPAA, Texas Administrative Codes 202-203, University of Texas Policy 165, federal regulations outlined in 21 CFR Part 11, and UTMDACC Institutional Policy #ADM0335.

Those having access to the data file will include the study chair and research team personnel. Users are authenticated against MDACC's Active Directory system. The application is accessed through Secure Socket Layer (SSL). Following publication study data will be archived in REDCap. Since study data may be useful for future research studies performed under separate IRB approved protocols, study data will be stored indefinitely in the REDCap database system [44] unless otherwise agreed upon in a clinical trial agreement (CTA) with participating site(s).

7. SAFETY AND ADVERSE EVENTS

7.1 ADVERSE EVENTS

An **AE** is the development of an undesirable medical condition or the deterioration of a pre-existing medical condition following or during exposure to the research, whether or not considered causally related to the research.

In this protocol, the **safety evaluation** will be performed based on the surgical complications that occurred during surgery and within 30 days after surgery (see Table 1). Adverse events related to chemotherapy administered to the patient after surgery will not be collected.

Adverse events of special interest (AESI) that require longer follow-up are defined in **Section 7.1.2.4** and **Table 1**.

All potential adverse events complications will be documented at the time of the next study visit.

7.1.1 SURGICAL AE

The surgical AE are defined in the list below.

- Intraoperative AE, which occur during surgical procedures
- Perioperative AE, which occur from day 1 to day 7 after surgery
- Postoperative AE, which occur from day 8 to 30 days after surgery

Possible complications of surgical procedures are listed below.

- Postoperative death (<30 days)
- Hemorrhage/bleeding intraoperative or postoperative requiring at least transfusion of 2 units non-autologous packed red blood cells (pRBCs) or major urgent interventions
- Vascular events: thrombosis/embolism, disabling or life-threatening vessel injury-artery or vein, symptomatic or life-threatening visceral arterial ischemia
- Infections requiring IV antibiotics, antifungal or antiviral interventions or at risk for life-threatening consequences
- Gastrointestinal fistula
- Gastrointestinal perforation
- Urinary fistula
- Neurological symptoms (anaesthesia, paraesthesia, dysesthesia)
- Wound healing
- Injury to abdominal or pelvic viscera secondary to endoscopic instrument placement or manipulation. This includes injury to the bowel, bladder, vessels, ureter or kidney

Intra-, peri- and postoperative adverse events of any grade will be rated using the CTC-AE (Version 5.0) and will be considered for statistical analysis. The rate of surgical AE will be compared between the two arms.

7.1.2 SAE

A SAE is an AE occurring during the study that fulfills one or more of the following criteria.

- Results in death
- Is immediately life-threatening
- Requires in-patient hospitalization or prolongation of existing hospitalization
- Results in persistent or significant disability/incapacity or substantial disruption of the ability to conduct normal life functions
- Is a congenital abnormality or birth defect
- Is an important medical event that may jeopardize the patient or may require medical intervention to prevent one of the outcomes listed above

The expression "life threatening" is defined as condition manifesting itself by acute symptoms of sufficient severity such that the absence of immediate medical attention could reasonably be expected to result in placing the individual's health in serious jeopardy, serious impairment to bodily functions, or serious dysfunction of bodily organs.

The terms "disability" and "incapacity" correspond to all physical or mental disabilities, either temporary or permanent, that are clinically relevant and with associated physical and/or quality of life consequences.

A "medically significant event" is defined as any clinical event or laboratory result judged as serious by the investigator or/and the protocol PI that does not fulfill the intensity criteria defined above. The patient cannot be put at risk by it or require medical intervention to prevent an issue corresponding to one of the intensity criteria defined above. For example, second primary cancers, intensive treatment in an emergency room can be considered as medically significant.

Events that do not meet the definition of a SAE include:

- Disease progression or death as a result of disease progression,
- Elective hospitalization for pre-existing conditions that have not been exacerbated by acts performed or methods used in the study,
- Hospitalization for <24 h or not judged by the investigator to be related to acts performed or methods used in the study, and/or
- Hospitalizations planned at the beginning of the study and / or provided in the protocol and/or progressive disease management or events occurring between informed consent signature and acts performed, and not related to protocol procedures.

7.1.2.1 EXPECTED SAE

An expected serious adverse event is an event that is mentioned in the information described in the protocol or information relative to the acts and methods used during the research.

7.1.2.2 UNEXPECTED SAE

An unexpected adverse event is an event for which the nature, the seriousness or the outcome is not consistent with the information relating to the practiced acts and to the methods used during the research.

7.1.2.3 NEW FACT

All new safety data which may lead to the re-evaluation of the risk-benefit balance of the research or of the acts performed or methods used, or that could be sufficient to consider modifications in the protocol.

7.1.2.4 ADVERSE EVENTS OF SPECIAL INTEREST (AESI)

The following AESIs will be followed up to 30 days post-surgery and up to 6 months post-surgery.

- Vascular events: thrombosis/embolism, disabling or life-threatening vessel injury-artery or vein, symptomatic or life-threatening visceral arterial ischemia
- Infections requiring IV antibiotics, antifungal or antiviral interventions or at risk for life-threatening consequences
- Gastrointestinal fistula
- Gastrointestinal perforation
- Urinary fistula
- Neurological symptoms (anaesthesia, paraesthesia, dysesthesia)
- Impaired wound healing
- Port site metastasis* for the MIS group (Port site metastasis is a rare event and the average time to occurrence following ovarian cancer MIS is unknown. Thus, this AESI will be collected until the end of the study.)

7.2 AE DESCRIPTION

7.2.1 TIME PERIOD FOR COLLECTION OF AES

All ongoing and any new AEs, AEs of Interest, and SAEs identified must be followed to resolution.

7.2.2 VARIABLES

Each AE should be evaluated to determine the following.

1. The severity of the grade (grade 1 to 5 according to CTCAE) .
2. The relationship with the study intervention. The investigators must do their best to explain each AE and establish whether there is a link between the study intervention (process, method, etc.) and the AE. The link cause and effect will be established in the following manner.
    - No, there is no reasonable causal relationship between the research and the AE.
    - There is a reasonable causal relationship between the research and the AE according to the following criteria:
        - The relationship between the study activities and the AE is known and/or
        - The effects are similar in nature to those that had been reported previously in the literature.
3. Whether the AE is a SAE

The following variables will be collected for each AE.

- AE (verbatim)
- The date when the AE started and stopped
- Changes in CTCAE
- Whether the AE is serious or not
- Investigator causality rating against the acts performed or methods used in the study (yes or no)
- Action taken with regard to acts performed or methods used in the study

When recording AEs, the recording of diagnosis is preferred to a list of signs and symptoms (when possible). However, if a diagnosis is known and there are other signs or symptoms that are not generally part of the diagnosis, the diagnosis and each sign or symptom will be recorded separately.

The results from protocol mandated laboratory tests and vital signs will be summarized in the clinical study report. Deterioration as compared to baseline in protocol-mandated laboratory values, vital signs should therefore only be reported as AEs if they fulfill any of the SAE criteria.

If deterioration in a laboratory value/vital sign/ECG is associated with clinical signs and symptoms, the sign or symptom will be reported as an AE and the associated laboratory result/vital sign/ECG will be considered as additional information. Wherever possible the reporting investigator uses the clinical, rather than the laboratory term (e.g., anemia versus low hemoglobin value). In the absence of clinical signs or symptoms, clinically relevant deteriorations in non-mandated parameters should be reported as AE(s).

Deterioration of a laboratory value, which is unequivocally due to disease progression, or chemotherapy should not be reported as an AE/SAE.

Any new or aggravated clinically relevant abnormal medical finding at a physical examination as compared with the baseline assessment will be reported as an AE.

7.2.2.1 SEVERITY OF AE

For each episode of an AE, only those attaining CTCAE grade ≥ 2 or clinically significant AE grade 1 will be reported. All changes to the CTCAE grade attained as well as the highest attained CTCAE grade should be reported.

It is important to distinguish between serious and severe AEs. Severity is a measure of intensity whereas seriousness is defined by the criteria in **Section** Error! Reference source not found.. An AE of severe intensity need not necessarily be considered as serious. For example, nausea that persists for several hours may be considered severe nausea, but not a SAE. On the other hand, a stroke that results in only a limited degree of disability may be considered as a mild stroke but would be an SAE (this would be recorded as an SAE as per the guidance found in this section).

The grading scales found in the National Cancer Institute (NCI) CTCAE version 5.0 will be utilized for all events with an assigned CTCAE grading. The CTCAE reports 1 to 5 grades with specific severity clinical description of each AE following the general procedures presented in **Table 4**.

Table 4. CTCAE Grade Definitions

CTC Grade	Equivalent to	Definition
Grade 1	Mild	• Asymptomatic or mild symptoms<br>• Clinical or diagnostic observations only<br>• Intervention not indicated
Grade 2	Moderate	• Moderate, minimal, local or non-invasive intervention indicated<br>• Limiting age-appropriate instrumental activity of daily living (ADL)
Grade 3	Severe	• Severe or medical significant but not immediately life-threatening<br>• Hospitalization or prolongation of hospitalization indicated<br>• Disabling<br>• Limiting self-care ADL
Grade 4	Life threatening	• Life-threatening consequences<br>• Urgent intervention indicated

Grade 5	Death	• Death related to AE

For those events without assigned CTCAE grades the recommendation is that the CTCAE criteria that convert mild, moderate and severe events into CTCAE grades should be used. Only moderate and severe events will be reported.

7.2.2.2 CAUSALITY COLLECTION

The site Investigator will assess causal relationship between acts performed or methods used in the study and each AE according to the degrees of causality presented in **Table 5**. In situations in which causal relationship is unclear, a third party committee of gynecologic oncologists will review and document relationship.

Table 5. Causality Grades and Evaluation Criteria

Imputability Grade	Evaluation criteria
Certain	• Clinical event or laboratory abnormalities, with plausible temporal relationship with the research<br>• Event that could not be explained by any illness or other drug<br>• Event identified by references of the research<br>• Positive re-challenge (where required)
Likely	• Clinical event or laboratory abnormalities, with reasonable temporal relationship with the research<br>• Does not appear to be related to any illness or other drug<br>• Re-challenge information required
Possible	• Clinical event or laboratory abnormalities, with reasonable temporal relationship with the research<br>• Does not appear to be related to any illness or other drug
Unlikely	• Clinical event or laboratory abnormalities, with improbable temporal relationship with the research<br>• Illness or other drug explaining plausible occurrence of the event

Note that for SAEs that could be associated with any study procedure, the causal relationship is implied as 'certain'.

7.3 REPORTING OF SAE

7.3.1 INITIAL NOTIFICATION

Only SAEs related to study procedures must be reported. All SAEs will be recorded in the CRF.

7.3.2 VARIABLES

In addition to the variables collected for each AE, the following variables will be collected for SAEs.

- Patient number
- Investigator identification and site number
- Previous and current medical history
- Date AE met criteria for serious AE

- Date investigator became aware of serious AE
- Date of hospitalization
- Date of discharge
- Outcome
- If the acts performed or methods used in the study have been discontinued
- Concomitant treatment
- Measures taken and need for a corrective treatment
- Probable cause of death
- Date of death
- Autopsy performed or not
- Causality assessment in relation to study procedure(s)
- Causality assessment in relation to other medication
- Description of AE

If possible, the investigator must attach to the SAE form the following documents.

- A copy of the hospitalization or prolongation of hospitalization report
- A copy of all additional exams realized, including relevant results containing laboratory normal values
- All judged relevant documents
- A copy of the final autopsy report

7.3.3 SAE FOLLOW UP

The investigator will be assuming the appropriate medical follow-up until resolution or stabilization of the effect or until the patient death. This could mean a prolonged follow-up after patient withdrawal from study.

7.4 DEATH NOTIFICATION

All deaths that **occur as a result from the surgery** must be reported according to the following guidelines.

- Death as clearly the result of disease progression should be reported to the study monitor at the next monitoring visit and should be documented in the “Death” section of the eCRF but should not be reported as a SAE.
- Where death is not due (or not clearly due) to progression of the disease under study, the AE causing the death must be reported to the study monitor as a SAE. The report should contain a comment regarding the co-involvement of progression of disease, if appropriate, and should assign main and contributory causes of death. This information can be collected in the “Death” section of the eCRF.
- Deaths with an unknown cause should always be reported as a SAE. A post mortem exam may be helpful in the assessment of the cause of death.

8. REFERENCES

1. Ferlay J, et al. Estimates of worldwide burden of cancer in 2008: GLOBOCAN 2008. International journal of cancer. Int J Cancer. 2010;127(12):2893-917.
2. Torre LA, Bray F, Siegel RL, et al. Global cancer statistics, 2012. CA Cancer J Clin 2015; 65:87-108.
3. Prat J, FIGO Committee on Gynecologic Oncology. Staging classification for cancer of the ovary, fallopian tube, and peritoneum. Int J Gynaecol Obstet. 2014; 124(1):1.
4. Cannistra SA. Cancer of the ovary. N Engl J Med 2004; 351: 2519-2529.
5. Lin JJ, Egorova N, Franco R, Prasad-Hayes M, Bickell NA. Ovarian Cancer Treatment and Survival Trends Among Women Older Than 65 Years of Age in the United States, 1995-2008. Obstet Gynecol 2016; 127(1): 81-9.
6. Harries M, Gore M. Part I: chemotherapy for epithelial ovarian cancer-treatment at first diagnosis. Lancet Oncol. 2002; 3(9): 529-36.
7. Schorge JO, Clark RM, Lee SI, Penson RT. Primary debulking surgery for advanced ovarian cancer: are you a believer or a dissenter? Gynecol Oncol 2014; 135(3): 595-605.
8. Aletti GD, Dowdy SC, Gostout BS, et al. Aggressive surgical effort and improved survival in advanced-stage ovarian cancer. Obstet Gynecol. 2006;107: 77-85.
9. Fader AN, Rose PG. Role of surgery in ovarian carcinoma. J Clin Oncol. 2007; 25(20): 2873-83.
10. Holdschneider CH, Sehouli, Karlan BY. Cytoreductive surgery: principles and rationale. In: Bristow RE, Karlan BY, Chi DS, editors. Surgery for Ovarian Cancer: Principles and Practices, 3rd ed. Boca Raton: CRC Press, 2016. p105-128.
11. Hacker NF, Berek JS. Cytoreductive surgery for ovarian cancer. In: Albert DS, Surwit EA editors. Ovarian Cancer. The Hague: Martinus Nijhoff, 1985. p 53-67.
12. Schorge JO, Bradford LS, del Carmen MG. Primary cytoreductive surgery for advanced ovarian cancer: is it the past, present, or future? Clin Adv Hematol Oncol 2011; 9(12): 912-8.
13. Vergote I, Tropé CG, Amant F, Ehlen T, Reed NS, Casado A. Neoadjuvant chemotherapy is the better treatment option in some patients with stage IIIc to IV ovarian cancer. J Clin Oncol. 2011; 29(31): 4076-8
14. Rauh-Hain JA, Rodriguez N, Growdon WB, Goodman AK, et al. Primary debulking surgery versus neoadjuvant chemotherapy in stage IV ovarian cancer. Ann Surg Oncol 2012; 19(3): 959-65.
15. Vergote I, Tropé CG, Amant F, Kristensen GB, Ehlen T, Johnson N, et al. Neoadjuvant chemotherapy or primary surgery in stage IIIC or IV ovarian cancer. N Engl J Med. 2010; 363:943-53.
16. Kehoe S, Hook J, Nankivell M, Jayson GC, Kitchener H, Lopes T, et al. Primary chemotherapy versus primary surgery for newly diagnosed advanced ovarian cancer (CHORUS): an open-label, randomised, controlled, non-inferiority trial. Lancet. 2015; 386:249-57.
17. Qin M, Jin Y, Ma L, et al. The role of neoadjuvant chemotherapy followed by interval debulking surgery in advanced ovarian cancer: a systematic review and meta-analysis of randomized controlled trials and observational studies. Oncotarget. 2017; 9(9):8614-8628.
18. Melamed A, Hinchcliff EM, Clemmer JT, Bregar AJ, Uppal S, Bostock I, Schorge JO, Del Carmen MG, Rauh-Hain JA. Trends in the use of neoadjuvant chemotherapy for advanced ovarian cancer in the United States. Gynecol Oncol. 2016; 143(2):236-240.
19. Wright AA, Bohlke K, Armstrong DK, Bookman MA, Cliby WA, et al. Neoadjuvant Chemotherapy for Newly Diagnosed, Advanced Ovarian Cancer: Society of Gynecologic Oncology and American Society of Clinical Oncology Clinical Practice Guideline. J Clin Oncol.

2016; 34(28): 3460-73.

20. Walker JL, Piedmonte MR, Spirtos NM, et al. Laparoscopy compared with laparotomy for comprehensive surgical staging of uterine cancer: Gynecologic Oncology Group Study LAP2. J Clin Oncol 2009; 27: 5331-5336.
21. Janda M, Gebski V, Davies LC, Forder P, et al. Effect of Total Laparoscopic Hysterectomy vs Total Abdominal Hysterectomy on Disease-Free Survival Among Women With Stage I Endometrial Cancer: A Randomized Clinical Trial. JAMA. 2017; 317(12): 1224-1233.
22. Ramirez PT, Frumovitz M, Pareja R, Lopez A, et al. Minimally Invasive versus Abdominal Radical Hysterectomy for Cervical Cancer. N Engl J Med. 2018; 379(20): 1895-1904.
23. Melamed A, Margul DJ, Chen L, Keating NL, et al. Survival after Minimally Invasive Radical Hysterectomy for Early-Stage Cervical Cancer. N Engl J Med. 2018; 379(20): 1905-1914.
24. Melamed A, Keating NL, Clemmer JT, Bregar AJ, et al. Staging for Apparent Stage I Epithelial Ovarian Cancer: Analysis of the National Cancer Data Base. Am J Obstet Gynecol. 2017; 216(1): 50.e1-50.e12.
25. Nezhat FR, Pejovic T, Finger TN, Khalil SS. Role of minimally invasive surgery in ovarian cancer. J Minim Invasive Gynecol 2013; 20: 754-765.
26. Gueli Alletti S, Bottoni C, Fanfani F, et al. Minimally invasive interval debulking surgery in ovarian neoplasm (MISSION trial-NCT02324595): a feasibility study. Am J Obstet Gynecol. 2016; 214: 503.e1-6.
27. Corrado G, Mancini E, Cutillo G, et al. Laparoscopic Debulking Surgery in the Management of Advanced Ovarian Cancer After Neoadjuvant Chemotherapy. Int J Gynecol Cancer.2015; 25(7): 1253-1257.
28. Favero G, Macerox N, Pfiffer T, et al. Oncologic concerns regarding laparoscopic cytoreductive surgery in patients with advanced ovarian cancer submitted to neoadjuvant chemotherapy. Oncology 2015; 89(3): 159-166.
29. Melamed A, Nitecki R, Boruta DM, del Carmen MG, et al. Laparoscopy Compared With Laparotomy for Debulking Ovarian Cancer After Neoadjuvant Chemotherapy. Obstet Gynecol. 2017; 129(5): 861-869.
30. Eisenhauer EA, Therasse P, Bogaerts J, et al. New response evaluation criteria in solid tumours: revised RECIST guideline (version 1.1). Eur J Cancer 2009; 45: 228-247.
31. Rustin GJ, Marples M, Nelstrop AE, Mahmoudi M, Meyer T. Use of CA-125 to define progression of ovarian cancer in patients with persistently elevated levels. J Clin Oncol 2001; 19: 4054-4057.
32. Dindo D, Demartines N, Clavien P. Classification of surgical complications: a new proposal with evaluation in a cohort of 6336 patients and results of a survey. Ann.Surg. 2004; 240: 205-13.
33. Armstrong, DK, Bundy, B, Wenzel L, the Gynecologic Oncology Group. Intraperitoneal cisplatin and paclitaxel in ovarian cancer. N Engl J Med. 2006; 354: 34-43.
34. Katsumata, N, Yasuda, M, Takahashi F, and the Japanese Gynecologic Oncology Group. Dose-dense paclitaxel once a week in combination with carboplatin every 3 weeks for advanced ovarian cancer: a phase 3, open-label, randomised controlled trial. Lancet. 2009; 374: 1331-1338.
35. Pignata S, Scambia G, Katsaros D, Gallo C, et al. Carboplatin plus paclitaxel once a week versus every 3 weeks in patients with advanced ovariancancer (MITO-7): a randomised, multicentre, open-label, phase 3 trial. Lancet Oncol. 2014; 15(4): 396-405.
36. Ozols RF, Bundy BN, Greer BE, Fowler JM, et al. Phase III trial of carboplatin and paclitaxel compared with cisplatin and paclitaxel in patients with optimally resected stage III ovarian cancer: a Gynecologic Oncology Group study. J Clin Oncol. 2003; 21(17): 3194-200.
37. Rouzier R, Gouy S, Selle F, Lambaudie E, et al. Efficacy and safety of bevacizumab-containing

neoadjuvant therapy followed by interval debulking surgery in advanced ovarian cancer: Results from the ANTHALYA trial. Eur J Cancer. 2017; 70:133-142.

38. Burger, RA, Brady, MF, Bookman, MA et al. Incorporation of bevacizumab in the primary treatment of ovarian cancer. N Engl J Med 2011; 365: 2473– 83.
39. Perren, TJ, Swart, AM, Pfisterer, J et al. A phase 3 trial of bevacizumab in ovarian cancer. N Engl J Med 2011; 365: 2484– 96.
40. Moore K, Colombo N, Scambia G, Kim BG, et al. Maintenance Olaparib in Patients with Newly Diagnosed Advanced Ovarian Cancer. N Engl J Med. 2018 27;379(26):2495-2505.
41. Stark D, Nankivell M, Pujade-Lauraine E, Kristensen G, et al. Standard chemotherapy with or without bevacizumab in advanced ovarian cancer: quality-of-life outcomes from the International Collaboration on Ovarian Neoplasms (ICON7) phase 3 randomised trial. Lancet Oncol. 2013; 14(3): 236-43.
42. EORTC Quality of Life, EORTC QLQ-C30. http://groups.eortc.be/qol/eortc-qlq-c30
43. Brookmeyer R, Crowley J. A confidence interval for the median survival time. Biometrics 1982; 38: 29-41.
44. Harris, P.A., et al., Research electronic data capture (REDCap)--a metadata-driven methodology and workflow process for providing translational research informatics support. J Biomed Inform,2009. 42(2): p. 377-81.
