# Supplementary material for: Laparoscopic Cytoreduction After Neoadjuvant Chemotherapy in High-Grade Epithelial Ovarian Cancer: A LANCE Randomized Clinical Trial
Source: JAMA Netw Open. 2024 Nov 21;7(11):e2446325. doi: 10.1001/jamanetworkopen.2024.46325 (PMC11582931; doi:10.1001/jamanetworkopen.2024.46325)
Supplement: Supplement 2. — eMethods. Power Calculations eTable. Postoperative Complications by Grade and Treatment eReferences [file jamanetwopen-e2446325-s002.pdf]

Supplemental Online Content

Rauh-Hain JA, Melamed A, Pareja R, et al. Laparoscopic cytoreduction after neoadjuvant chemotherapy in high-grade epithelial ovarian cancer: a LANCE randomized clinical trial. *JAMA Netw Open*. 2024;7(11):e2446325. doi:10.1001/jamanetworkopen.2024.46325

eMethods. Power Calculations

eTable. Postoperative Complications by Grade and Treatment

eReferences

This supplemental material has been provided by the authors to give readers additional information about their work.

© 2024 Rauh-Hain JA et al. *JAMA Network Open*

eMethods. Power Calculations

We set a putative sample size of 100 for the lead-in pilot phase of the study. For each co-endpoint, we investigated the type I and II error rates achievable with this sample size. We determined that it would be possible to achieve reasonable power (> 70%) for the accrual rate co-endpoint with a 1-sided study-wise type I error rate of 0.2.[1] Fixing the type I error rate at 0.2 for the other co-primary end points was compatible with achieving very high power (> 98%) for each of them and allowed us to maintain a reasonable overall type II error rate. Since endpoints were co-primary, no adjustment of α was required for multiplicity.[2] We estimated the feasibility study's overall power in a simulation study.

The accrual endpoint was defined as the accrual rate estimated by fitting a LOWESS curve with the monthly recruitment rate as the dependent variable and a month since the study inception as the dependent variable. We then compared the predicted rate in the final completed calendar month of the lead-in pilot study with a threshold value. We conducted simulations to characterize the sample size characteristics of the accrual endpoint.[3] First, we simulated 5000 studies in which the true accrual rate (d') increased linearly from the time of the study initiation, reached 7 patients per month after 12 months, and remained constant until 100 patients were accrued (Supplementary Figure 1). In each simulation, the monthly enrollment was simulated by drawing from a Poisson

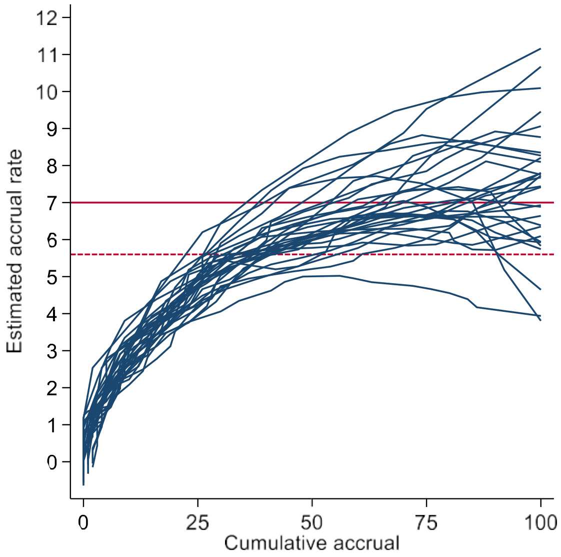

Supplementary Figure 1. Thirty simulations of accrual. The solid red line represents the actual accrual rate reached by 12 months. The dashed red line is the accrual threshold for feasibility.

© 2024 Rauh-Hain JA et al. *JAMA Network Open*

distribution in which $\lambda = d''$. The estimated accrual rate in the final month was calculated for each simulation study from the LOWESS curve. A feasibility threshold of 5.6 patients per month corresponded to 77.7% power to detect an enrollment rate of 7 patients per month. By varying the final value of $\ddot{a}$ in an additional 5000 simulation studies, we determined that, using a feasibility threshold value of 5.6 patients per month, the lead-in phase would incorrectly reject the null hypothesis of an enrollment rate of 4.5 patients per month or less in 20% of simulations (1-sided $\alpha = 0.2$).

Assuming a 1-sided $\alpha = 0.2$, 50 patients in the MIS arm, and a crossover rate of 32% under the null hypothesis, a feasibility threshold value with less than 25% crossover in the MIS group has 98.7% power to detect a crossover rate of 15% or less.[4] With 100 equally allocated subjects, a 1-sided α of 0.2, and a noninferiority margin of 27.0 percentage points, a feasibility threshold value with less than 20 percentage points difference in the complete gross resection rate between the 2 study arms has 99.9% power to reject the null hypothesis of inferiority when the true rate of complete gross resection is 90% in both study arms.[5]

The threshold values for each co-primary endpoint were designed to achieve a type I error rate of 0.2 for each endpoint, leading to a study-wise type I error rate of 0.2 if feasibility is established by rejecting all null hypotheses simultaneously. To estimate the power of the overall study, we simultaneously simulated accrual (drawing from the Poisson distribution as described above), crossover in the MIS arm (drawing from a binomial distribution), and the rates of optimal gross resection in each arm (also drawn from binomial distributions) for 5000 feasibility studies under the alternative hypothesis for all endpoints. In these simulations, simultaneous feasibility for all 3 endpoints was achieved in 75.4% of studies. These simulations assume the independence of the co-primary endpoints.

© 2024 Rauh-Hain JA et al. *JAMA Network Open*

eTable. Postoperative Complications by Grade and Treatment

	Grade	CTCAE grade I	II	III	IV	V	
	**Complication type**						**Total**
Open	Respiratory	1	1	0	0	0	2
	Gastrointestinal and liver	3	0	0	1	0	4
	Renal and genitourinary	4	1	0	0	0	5
	Wound	0	3	0	0	0	3
	Hematologic	0	8	0	0	0	8
	Infection	0	0	1	0	0	1
	Total	8	13	1	1	0	23
MIS	Respiratory	1	0	0	0	0	1
	Gastrointestinal, Liver	1	0	0	1	0	2
	Renal and GU	3	1	0	0	0	4
	Sepsis	0	0	0	0	1	1
	Hematologic	0	3	1	0	0	4
	Total	5	4	1	1	1	12

Abbreviation: MIS, minimally invasive surgery. CTCAE: Common Terminology Criteria for Adverse Events

© 2024 Rauh-Hain JA et al. *JAMA Network Open*

eReferences

1. Schoenfeld D. Statistical considerations for pilot studies. *Int J Radiat Oncol Biol Phys.* 1980 Mar;6(3):371-4. doi: 10.1016/0360-3016(80)90153-4. PMID: 7390914.
2. Federal Drug Administration. Multiple endpoints in clinical trials guidance for industry. 2022. Available online at https://www.fda.gov/regulatory-information/search-fda-guidance-documents/multiple-endpoints-clinical-trials-guidance-industry. Accessed on Feb 6, 2024.
3. Arnold BF, Hogan DR, Colford JM Jr, Hubbard AE. Simulation methods to estimate design power: An overview for applied research. *BMC Med Res Methodol.* 2011;11:94. doi: 10.1186/1471-2288-11-94. PMID: 21689447; PMCID: PMC3146952.
4. Fleiss, JL. *Statistical Methods for Rates and Proportions* (1981), pp. 13-15.
5. Kopecky K and Green S (2012). Noninferiority trials. In: Handbook of Statistics in Clinical Oncology. Crowley J and Hoering A, eds. CRC Press, Boca Raton, FL, USA.
