# Supplementary material for: Laparoscopic Cytoreduction After Neoadjuvant Chemotherapy in High-Grade Epithelial Ovarian Cancer: A LANCE Randomized Clinical Trial
Source: JAMA Netw Open. 2024 Nov 21;7(11):e2446325. doi: 10.1001/jamanetworkopen.2024.46325 (PMC11582931; doi:10.1001/jamanetworkopen.2024.46325)
Supplement: Supplement 3. — Data Sharing Statement [file jamanetwopen-e2446325-s003.pdf]

Data Sharing Statement

Rauh-Hain. Laparoscopic Cytoreduction After Neoadjuvant Chemotherapy in High-Grade Epithelial Ovarian Cancer. *JAMA Netw Open*. Published November 21, 2024. doi:10.1001/jamanetworkopen.2024.46325

Data
Additional Information: Trial registration: Laparoscopic Cytoreduction After Neoadjuvant Chemotherapy (LANCE) trial (NCT04575935): https://clinicaltrials.gov/study/NCT04575935. ;
Data available: No

Additional Information
Explanation for why data not available: This is the feasibility phase of the study.
